# Design, Synthesis, and Evaluation of Cytotoxic Effects of Functional Fatty Acid Derivatives as Potential Antineoplastic Agents for Breast Cancer

**DOI:** 10.5812/ijpr-159523

**Published:** 2025-03-15

**Authors:** Maryam Hosseini, Farzad Kobarfard, Salimeh Amidi, Shaya Mokhtari, Anna Sedaghat, Soraya Shahhosseini

**Affiliations:** 1 Department of Medicinal Chemistry, School of Pharmacy, Shahid Beheshti University of Medical Sciences, Tehran, Iran; 2 Central Research Laboratories, Shahid Beheshti University of Medical Sciences, Tehran, Iran; 3 Department of Medicinal Chemistry, School of Pharmacy, Iran University of Medical Sciences, Tehran, Iran; 4 Department of Neurology, Division of Clinical Neuroscience, Oslo University Hospital, Oslo, Norway; 5 Department of Chemistry, University of Oslo, Blindern, Oslo, Norway

**Keywords:** Docosahexaenoic Acid, Linoleic Acid, Breast Cancer, Molecular Docking, MTT Assay, Flow Cytometry

## Abstract

**Background:**

Breast cancer is among the most prevalent cancers in women and is the leading cause of mortality among women worldwide. Although a definitive cure for breast cancer remains elusive, essential fatty acids offer a promising therapeutic avenue.

**Objectives:**

The present study aimed to synthesize 16 derivatives of docosahexaenoic acid (DHA) and linoleic acid (LA) and evaluate their anti-cancer properties in vitro.

**Methods:**

Fourteen derivatives of LA and DHA were synthesized using a coupling method, while two ethylenediamine derivatives were synthesized via an ester intermediate. Molecular modeling was conducted using AutoDock Vina software. The cytotoxic effects of all compounds were assessed using the MTT assay on breast adenocarcinoma (MCF-7) cells. The mechanism of cell death induction by derivatives with the most favorable EC_50_ values was determined through annexin V-FITC/PI flow cytometry analysis, focusing on early and late apoptosis.

**Results:**

Docking results revealed that these compounds effectively interact with residues in the PTPB1 active site. All synthesized DHA and LA derivatives demonstrated cytotoxic effects on the MCF-7 cell line, with no significant cytotoxicity observed in normal human dermal fibroblasts (HDFs). Compounds D3 and L3, with EC_50_ values of 15.96 ± 2.89 μM and 24.64 ± 1.81 μM, respectively, were identified as the most potent anti-cancer compounds among the derivatives.

**Conclusions:**

The findings indicate that these functional fatty acid derivatives significantly reduce cancer cell viability. In addition to necrosis, compounds L3 and D3 induced apoptosis, with apoptosis rates of 20.5% and 47.1%, respectively.

## 1. Background

The currently approved chemotherapy drugs are not completely effective in cancer treatment, and new drugs with novel mechanisms are under development. This limitation, along with late diagnosis, has ranked cancer as the second leading cause of death worldwide (1). Consequently, early diagnosis and effective treatments have become key priorities for researchers. Tumor-targeting ligands are essential for developing highly specific anti-cancer drugs and enhancing the effectiveness of tumor imaging agents (2-4). The incidence rate of female breast cancer is projected to be 129.4 new cases per 100,000 women annually. The age-adjusted death rate is 19.3 per 100,000 women annually, based on case report data from 2017 - 2021 and 2018 - 2022 mortality records. In 2024, it is estimated that approximately 310,720 women and 2,800 men will be diagnosed with invasive breast cancer. In the United States, approximately 12.5% of women are diagnosed with breast cancer during their lifetime (5).

Genetic, environmental, hormonal, and dietary factors all contribute to the development of breast cancer. Long-chain fatty acids are considered one of the dietary factors linked to breast cancer risk. However, their exact role in either promoting or preventing the progression of breast cancer is not fully understood and remains controversial (6). The intake of long-chain fatty acids may reduce tumor growth by initiating certain biological processes, such as inducing apoptosis or inhibiting angiogenesis. Additionally, they may enhance the efficacy of chemotherapy drugs and reduce the side effects associated with chemotherapy or cancer treatments, while being associated with a high level of safety (7-11).

For many cancers, conventional medicines have poor success rates. Therefore, improving the understanding of disease progression and exploring alternative therapeutics is required. Long-chain polyunsaturated fatty acids (PUFAs), such as docosahexaenoic acid (DHA) and linoleic acid (LA), have been shown to reduce cancer cell survival and inhibit their proliferation both in vitro and in vivo. Docosahexaenoic acid, a natural omega-3 fatty acid with 22 carbon atoms and six cis double bonds, has been shown to induce apoptosis and reduce proliferation in cancerous cells both in vitro and in vivo (1, 12, 13). Nevertheless, there is a lack of research on the effect of DHA derivatives on cancer cell apoptosis, and their mechanisms of action are not fully recognized (1, 12-14). Reviews on DHA metabolites in living systems indicate that numerous and diverse compounds are formed in cells, which have various vital activities. Resolvins, protectins, and maresins are some of these DHA metabolites that play a central role in controlling inflammation (15).

Recent research has revealed that ingested DHA and eicosapentaenoic acid (EPA) are metabolized into their corresponding ethanolamide metabolites, docosahexaenoylethanolamide (DHEA) and eicosapentaenoylethanolamide (EPEA) (16-18). These metabolites act as lipid mediators both in vitro and in vivo (19, 20). It appears that at least part of the numerous and diverse reported roles for DHA and EPA in biological systems are due to their metabolites. DHEA and EPEA play an essential role in mediating the wide range of beneficial effects associated with DHA and EPA. Therefore, these ethanolamide metabolites show strong potential as candidate compounds for drug development, particularly for addressing diseases linked to omega-3 fatty acids (6, 17, 19-23).

Some tyrosine kinases, such as human epidermal growth factor receptor 1 (HER1/EGFR), SRC, signal transducer and activator of transcription (STAT), and JAK, are dephosphorylated by protein tyrosine phosphatase 1B (PTP1B). The PTP1B is found to be overexpressed in breast cancer cells (24) and contributes to tumor growth (25). Therefore, it is a critical regulator of signal transduction, particularly in relation to tyrosine kinase signaling pathways. According to virtual studies on DHA with enzyme PTP1B, DHA is able to interact with an allosteric binding site, reducing both PTP1B enzyme activity and the viability of breast adenocarcinoma (MCF-7) cancer cells (26). Investigations have demonstrated that omega-3 fatty acids, when applied to MCF-7 cancer cells as an in vitro model, inhibit cell growth, promote differentiation, and induce apoptosis (27).

Apoptosis regulates cell populations during development and aging. It serves as a protective mechanism, allowing damaged cells to self-destruct and form apoptotic bodies without causing inflammation or harming their environment when encountering pathogens and toxic chemicals. The resulting apoptotic bodies are cleared by phagocytosis (28).

## 2. Objectives

In the present study, DHA and LA amide derivatives were synthesized, and the anti-tumor effect of these fatty acids was investigated, focusing on the apoptosis induced by them on MCF-7 human breast cancer cells. This was investigated using the MTT assay and flow cytometry. A better understanding of the anti-tumor mechanism of functional fatty acids may help develop new strategies in cancer prevention and treatment, which involves incorporating fish oil as a nutritional additive.

## 3. Methods

### 3.1. Materials and Methods

All chemicals and solvents were obtained from Sigma-Aldrich and Merck AG as synthesis grade. When required, solvents were dried using standard procedures. A Cary 630 FTIR spectrometer was used to obtain the Fourier transform infrared (FTIR) spectrum. ^1^H-NMR spectra were recorded using a Bruker FT-400 MHz instrument (Bruker Biosciences, USA). Deuterated chloroform (CDCl_3_) and dimethyl sulfoxide (DMSO)-d6 were employed as solvents. Splitting patterns are denoted as singlet (s), doublet (d), triplet (t), quartet (q), broad singlet (bs), and multiplet (m). For liquid chromatography-mass spectrometry (LC-MS), a 6410 Agilent LC-MS triple quadrupole mass spectrometer equipped with an electrospray ionization (ESI) interface was used. Elemental analysis was performed using a Costech (Italy) elemental analyzer. Thin-layer chromatography (TLC) on 3 × 6 cm, 0.25 mm silica gel 60-F plates was used to monitor the reactions. Final docking images were generated using Discovery Studio 4.5 Visualizer and the PyMOL program (version 0.99rc6). Flow cytometric analyses were performed using flow cytometry (BD FACSCalibur, BD Biosciences, USA), and flow data were analyzed with FlowJo v.7.6.5 (Tree Star, Inc., Ashland, USA).

### 3.2. General Experimental Approach

1-Ethyl-3-(3-dimethylaminopropyl) carbodiimide (EDCI) (1.2 mmol) was added to a solution of DHA (1 mmol), 4-dimethylaminopyridine (DMAP) (1.2 mmol), and 2-aminoethanol (2 mmol) in dry dichloromethane (7 mL), and the mixture was stirred under a nitrogen atmosphere at room temperature overnight. The product was extracted with ethyl acetate (2 × 50 mL). Hydrochloric acid (15 mL, 1 N) and magnesium sulfate were used to wash and dry the combined organic layers, respectively (29). After evaporating the solvent, the obtained residue was purified using silica gel TLC (chloroform/methanol = 10:1) to afford compounds D1-D7 and L1-L7 in 68 - 85% yield.

**Figure 1.**
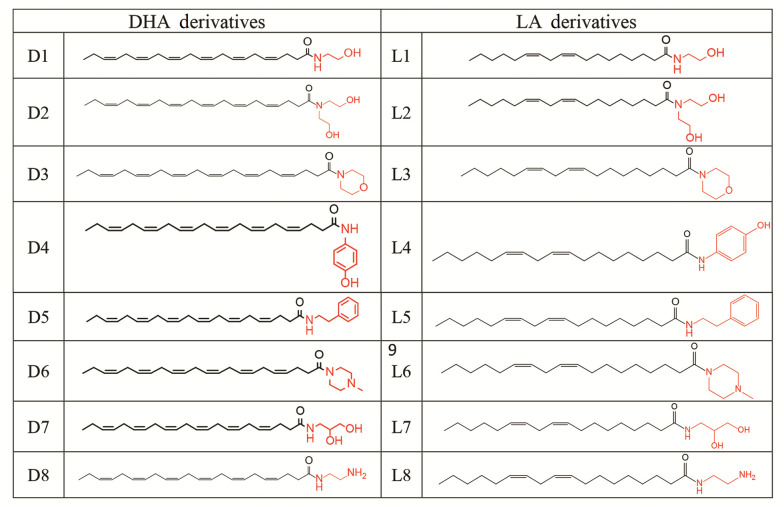
Amide derivatives of unsaturated fatty acids

Amide compounds D8 and L8 were synthesized by refluxing excess amounts of ethylenediamine (10 mmol) with a fatty acid methyl ester (1 mmol) in dry pyridine (5 mL) for 24 hours (30). The final solution was poured into cold water, and the insoluble crude product was purified using plate chromatography as explained above. Fatty acid methyl ester was prepared by a well-described method (31-34). Sixteen amides were prepared (Figure 1). 

#### 3.2.1. (4Z,7Z,10Z,13Z,16Z,19Z)-N-(2-hydroxyethyl) docosa-4,7,10,13,16,19-hexaenamide (D1)

Yield: 77%; Appearance: Yellow oil; IR (ν, cm^−1^): 1631 (C=O), 3317 (O-H); ^1^HNMR (DMSO-d6, 400 MHz): δ ppm 0.902 - 0.940 (t, 3H, CH_3_), 2.000 - 2.055 (m, 2H, CH_2_), 2.085 - 2.124 (t, 2H, CH_2_), 2.222 - 2.272 (m, 2H, CH_2_), 2.837 - 2.869 (m, 10H, CH_2_), 3.426 - 3.467 (m, 2H, CH_2_), 3.738 - 3.760 (t, 2H, CH_2_), 4.682 (s, 1H, OH), 5.351 - 5.439 (m, 12H, CH), 7.814 - 7.840 (s, 1H, NH); LC-MS (ESI) m/z: 394 (M+23); Anal. Calcd for C_24_H_37_NO_2_: C, 77.58; H, 10.04; N, 3.77; Found: C, 77.56; H, 10.01; N, 3.79.

#### 3.2.2. (4Z,7Z,10Z,13Z,16Z,19Z)-N,N-bis(2-hydroxyethyl)docosa-4,7,10,13,16,19-hexaenamide (D2)

Yield: 70%; Appearance: Yellow oil; IR (ν, cm^−1^): 1643 (C=O), 3414 (O-H); ^1^HNMR (CDCl3): δ ppm 0.965 - 1.003 (t, 3H, CH_3_), 2.044 - 2.105 (q, 2H, CH_2_), 2.378 - 2.437 (m, 4H, CH_2_), 2.825 - 2.859 (m, 10H, CH_2_), 3.215 - 3.293 (m, 3H, CH_2_), 3.568 - 3.592 (t, 1H, CH_2_), 3.780 - 3.878 (m, 4H, CH_2_), 4.146 (bs, 2H, OH), 5.380 - 5.392 (m, 12H, CH); LC-MS (ESI) m/z: 416 (M+1); Anal. Calcd for C_26_H_41_NO_3_: C, 75.14; H, 9.94; N, 3.37; Found: C, 75.16; H, 9.92; N, 3.39.

#### 3.2.3. 4-((4Z,7Z,10Z,13Z,16Z,19Z)-docosa-4,7,10,13,16,19-hexaen-1-yl)morpholine (D3)

Yield: 85%; Appearance: Yellow oil; IR (ν, cm^−1^): 1630 (C=O); ^1^HNMR (CDCl3): δ ppm 0.972 - 1.010 (t, 3H, CH_3_), 2.057 - 2.131 (m, 2H, CH_2_), 2.411 (bs, 4H, CH_2_), 2.819 - 2.864 (m, 10H, CH_2_), 3.134 - 3.173 (t, 1H, CH_2_), 3.420 - 3.493 (m, 2H, CH_2_), 3.646 - 3.728 (m, 4H, CH_2_), 3.880 - 3.906 (t, 1H, CH_2_), 5.299 - 5.414 (m, 12H, CH); LC-MS (ESI) m/z: 398 (M+1), 436 (M+39); Anal. Calcd for C_26_H_39_NO_2_: C, 78.54; H, 9.89; N, 3.52; Found: C, 78.57; H, 9.87; N, 3.51.

#### 3.2.4. (4Z,7Z,10Z,13Z,16Z,19Z)-N-(4-hydroxyphenyl)docosa-4,7,10,13,16,19-hexaenamide (D4)

Yield: 83%; Appearance: Yellow oil; IR (ν, cm^−1^): 1654 (C=O), 3302 (O-H); ^1^HNMR (DMSO-d6, 400 MHz): δ ppm 0.898 - 0.936 (t, 3H, CH_3_), 1.995 - 2.067 (m, 2H, CH_2_), 2.287 - 2.346 (m, 4H, CH_2_), 2.797 - 2.835 (m, 10H, CH_2_), 5.308 - 5.375 (m, 12H, CH), 6.654 - 6.675 (d, 2H, CH), 7.340 - 7.359 (d, 2H, CH), 7.963 (s, 1H, OH), 9.635 (s, 1H, NH); LC-MS (ESI) m/z: 418 (M-1); Anal. Calcd for C_28_H_37_NO_2_: C, 80.15; H, 8.89; N, 3.34; Found: C, 80.10; H, 8.91; N, 3.35.

#### 3.2.5. (4Z,7Z,10Z,13Z,16Z,19Z)-N-phenethyldocosa-4,7,10,13,16,19-hexaenamide (D5)

Yield: 80%; Appearance: Yellow oil; IR (ν, cm^−1^): 1647 (C=O), 3288 (N-H); ^1^HNMR (DMSO-d6, 400 MHz): δ ppm 0.825 - 0.863 (t, 3H, CH_3_), 1.939 - 2.025 (m, 4H, CH_2_), 2.145 - 2.194 (m, 2H, CH_2_), 2.602 - 2.634 (t, 2H, CH_2_), 2.707 - 2.762 (m, 10H, CH_2_), 3.153 - 3.204 (m, 2H, CH_2_), 5.221 - 5.272 (m, 12H, CH), 7.110 - 7.131 (d, 3H, CH), 7.188 - 7.223 (t, 2H, CH), 7.821 - 7.849 (t, 1H, NH); LC-MS (ESI) m/z: 432 (M+1), 454 (M+23); Anal. Calcd for C_30_H_41_NO: C, 83.47; H, 9.57; N, 3.24; Found: C, 83.44; H, 9.59; N, 3.25.

#### 3.2.6. (4Z,7Z,10Z,13Z,16Z,19Z)-1-(4-methylpiperazin-1-yl) docosa-4,7,10,13,16,19-hexaen-1-one (D6)

Yield: 77%; Appearance: Yellow oil; IR (ν, cm^−1^): 1655 (C=O); ^1^HNMR (DMSO-d6, 400 MHz): δ ppm 0.833 - 0.871 (t, 3H, CH_3_), 1.930 - 2.002 (m, 2H, CH_2_), 2.098 (s, 3H, CH_3_), 2.134 - 2.281 (m, 8H, CH_2_), 2.715 - 2.758 (m, 10H, CH_2_), 3.331 - 3.354 (t, 4H, CH_2_), 5.267 - 5.278 (m, 12H, CH); LC-MS (ESI) m/z: 411 (M+1); Anal. Calcd for C_27_H_42_N_2_O: C, 78.97; H, 10.31; N, 6.82; Found: C, 78.95; H, 10.30; N, 6.85.

#### 3.2.7. (4Z,7Z,10Z,13Z,16Z,19Z)-N-(2,3-dihydroxypropyl) docosa-4,7,10,13,16,19-hexaenamide (D7)

Yield: 85%; Appearance: Yellow oil; IR (ν, cm^−1^): 1653 (C=O), 3270 (N-H), 3398 (O-H); ^1^HNMR (CDCl3): δ ppm 0.958 - 0.996 (t, 3H, CH_3_), 2.054 - 2.108 (m, 2H, CH_2_), 2.178 - 2.289 (m, 2H, CH_2_), 2.302 - 2.314 (m, 2H, CH_2_), 2.793 - 2.879 (m, 10H, CH2), 3.313 - 3.383 (m, 2H, CH_2_), 3.449 - 3.534 (m, 2H, CH_2_), 3.703 - 3.739 (m, 1H, CH), 5.336 - 5.346 (m, 12H, CH), 7.938 (s, 1H, NH); LC-MS (ESI) m/z: 424 (M+23); Anal. Calcd for C_25_H_39_NO_3_: C, 74.77; H, 9.79; N, 3.49; Found: C, 74.79; H, 9.76; N, 3.48.

#### 3.2.8. (4Z,7Z,10Z,13Z,16Z,19Z)-N-(2-aminoethyl) docosa-4,7,10,13,16,19-hexaenamide (D8)

Yield: 59%; Appearance: Yellow oil; IR (ν, cm^−1^): 1632 (C=O), 3295 (N-H); ^1^HNMR (CDCl3): δ ppm 0.908-0.941 (t, 3H, CH3), 1.840 (s, 2H, NH2), 2.092-2.148 (m, 4H, CH2), 2.197-2.272 (m, 2H, CH2), 2.602-2.633 (t, 2H, CH2), 2.784-2.831 (m, 10H, CH2), 3.074-3.118 (m, 2H, CH2), 5.315-5.352 (m, 12H, CH), 8.064 (t, 1H, NH); LC-MS (ESI) m/z: 371 (M+1); Anal. Calcd for C24H38N2O: C, 77.79; H, 10.34; N, 7.56; Found: C, 77.78; H, 10.31; N, 7.59.

#### 3.2.9. (9Z,12Z)-N-(2-hydroxyethyl) octadeca-9,12-dienamide (L1)

Yield: 83%; Appearance: Yellow oil; IR (ν, cm^−1^): 1647 (C=O), 3310 (O-H); ^1^HNMR (CDCl_3_): δ ppm 0.856 - 0.889 (t, 3H, CH_3_), 1.289 (m, 14H, CH_2_), 1.603 (m, 2H, CH_2_), 2.006 - 2.039 (t of d, 4H, CH_2_), 2.164 - 2.201 (t, 2H, CH_2_), 2.737 - 2.768 (t, 2H, CH_2_), 3.368 - 3.379 (t, 2H, CH_2_), 3.658 - 3.680 (t, 2H, CH_2_), 5.307 - 5.382 (m, 4H, CH), 6.545 (s, 1H, NH); LC-MS (ESI) m/z: 346 (M+1); Anal. Calcd for C_20_H_37_NO_2_: C, 74.25; H, 11.53; N, 4.33; Found: C, 74.28; H, 11.51; N, 4.34.

#### 3.2.10. (9Z,12Z)-N,N-bis(2-hydroxyethyl) octadeca-9,12-dienamide (L2)

Yield: 74%; Appearance: Yellow oil; IR (ν, cm^-1^): 1677 (C=O), 3414 (O-H); ^1^HNMR (CDCl_3_): δ ppm 0.891 - 0.926 (t, 3H, CH_3_), 1.272 - 1.409 (m, 14H, CH_2_), 1.620 - 1.655 (t, 2H, CH_2_), 2.040 - 2.093 (t of d, 4H, CH_2_), 2.305 - 2.343 (t, 2H, CH_2_), 2.774 - 2.806 (t, 2H, CH_2_), 3.519 (bs, 4H, -NCH_2_-), 3.687 (bs, 4H, -CH_2_-OH), 4.331 (s, 1H, OH), 5.027 (s, 1H, OH), 5.315-5.420 (m, 4H, CH); LC-MS (ESI) m/z: 368 (M+1); Anal. Calcd for C_22_H_41_NO_3_: C, 71.89; H, 11.24; N, 3.81; Found: C, 71.85; H, 11.25; N, 3.82.

#### 3.2.11. (9Z,12Z)-1-morpholinooctadeca-9,12-dien-1-one (L3)

Yield: 74%; Appearance: Yellow oil; IR (ν, cm^−1^): 1655 (C=O); ^1^HNMR (CDCl_3_): δ ppm 0.866 - 0.875 (t, 3H, CH_3_), 1.306 (bs, 14H, CH_2_), 1.608 (m, 2H, CH_2_), 2.027 (m, 4H, CH_2_), 2.281 - 2.296 (t, 2H, CH_2_), 2.756 (m, 2H, CH_2_), 3.446 (t, 3H, CH_2_), 3.603 - 3.648 (t, 5H, CH_2_), 5.328 (m, 4H, CH); LC-MS (ESI) m/z: 372 (M+23); Anal. Calcd for C_22_H_39_NO_2_: C, 75.59; H, 11.25; N, 4.01; Found: C, 75.61; H, 11.23; N, 4.02.

#### 3.2.12. (9Z,12Z)-N-(4-hydroxyphenyl) octadeca-9,12-dienamide (L4)

Yield: 82%; Appearance: Yellow oil; IR (ν, cm^−1^): 1647 (C=O), 3288 (N-H), 3370 (O-H); ^1^HNMR (CDCl_3_): δ ppm 0.884 - 0.913 (t, 3H, CH_3_), 1.313 (bs, 14H, CH_2_), 1.641 - 1.702 (m, 2H, CH_2_), 2.052 (bs, 4H, CH_2_), 2.283 - 2.362 (m, 2H, CH_2_), 2.768 - 2.799 (m, 2H, CH_2_), 5.332 - 5.385 (m, 4H, CH), 6.707 - 6.727 (d, 2H, CH), 7.192 - 7.212 (d, 2H, CH), 7.932 (s, 1H, NH); LC-MS (ESI) m/z: 410 (M+39); Anal. Calcd for C_24_H_37_NO_2_: C, 77.58; H, 10.04; N, 3.77; Found: C, 77.55; H, 10.06; N, 3.79.

#### 3.2.13. (9Z,12Z)-N-phenethyloctadeca-9,12-dienamide (L5)

Yield: 84%; Appearance: Yellow oil; IR (ν, cm^−1^): 1647 (C=O), 3288 (N-H); ^1^HNMR (CDCl_3_): δ ppm 0.892 - 0.926 (t, 3H, CH_3_), 1.276 - 1.391 (m, 14H, CH_2_), 1.590 - 1.623 (m, 2H, CH_2_), 2.042 - 2.093 (m, 4H, CH_2_), 2.115 - 2.153 (t, 2H, CH_2_), 2.776 - 2.855 (m, 4H, CH_2_), 3.521 - 3.570 (m, 2H, CH_2_), 5.318 - 5.411 (m, 4H, CH), 7.205 - 7.355 (m, 5H, CH); LC-MS (ESI) m/z: 384 (M+1); Anal. Calcd for C_26_H_41_NO: C, 81.41; H, 10.77; N, 3.65; Found: C, 81.44; H, 10.75; N, 3.56.

#### 3.2.14. (9Z,12Z)-1-(4-methylpiperazin-1-yl) octadeca-9,12-dien-1-one (L6)

Yield: 75%; Appearance: Yellow oil; IR (ν, cm^−1^): 1647 (C=O); ^1^HNMR (CDCl_3_): δ ppm 0.910 - 0.934 (t, 3H, CH_3_), 1.338 (bs, 14H, CH_2_), 1.619 - 1.666 (m, 2H, CH_2_), 2.044 - 2.078 (m, 4H, CH_2_), 2.319 - 2.350 (t, 2H, CH_2_), 2.501 (s, 3H, CH_3_), 2.649 (bs, 4H, CH_2_), 2.778 - 2.809 (t, 2H, CH_2_), 3.670 (bs, 2H, CH_2_), 3.815 (bs, 2H, CH_2_), 5.314 - 5.409 (m, 4H, CH); LC-MS (ESI) m/z: 363 (M+1); Anal. Calcd for C_23_H_42_N_2_O: C, 76.19; H, 11.68; N, 7.73; Found: C, 76.16; H, 11.69; N, 7.75.

#### 3.2.15. (9Z,12Z)-N-(2,3-dihydroxypropyl) octadeca-9,12-dienamide (L7)

Yield: 83%; Appearance: Yellow oil; IR (ν, cm^−1^): 1640 (C=O), 3317 (O-H); ^1^HNMR (DMSO-d6, 400 MHz): δ ppm 0.844 - 0.881 (t, 3H, CH_3_), 1.241 - 1.296 (m, 14H, CH_2_), 1.450 - 1.491 (m, 2H, CH_2_), 1.993 - 2.089 (m, 6H, CH_2_), 2.721 - 2.755 (t, 2H, CH_2_), 2.934 - 3.001 (m, 1H, CH_2_), 3.136 - 3.198 (m, 1H, CH_2_), 3.253 - 3.274 (m, 2H, CH_2_), 3.429 - 3.471 (m, 1H, OH), 5.272 - 5.381 (m, 4H, CH); LC-MS (ESI) m/z: 354 (M+1), 376 (M+23); Anal. Calcd for C_21_H_39_NO_3_: C, 71.34; H, 11.12; N, 3.96; Found: C, 71.36; H, 11.10; N, 3.94.

#### 3.2.16. (9Z,12Z)-N-(2-aminoethyl) octadeca-9,12-dienamide (L8)

Yield: 65%; Appearance: Yellow oil; IR (ν, cm^−1^): 1632 (C=O), 3260, 3350; ^1^HNMR (CDCl_3_): δ ppm 0.773 - 0.806 (t, 3H, CH_3_), 1.172 - 1.242 (m, 14H, CH_2_), 1.364 - 1.435 (m, 2H, CH_2_), 1.765 (s, 2H, NH_2_), 1.923 - 1.985 (m, 6H, CH_2_), 2.652 - 2.682 (t, 2H, CH_2_), 2.941 - 2.987 (m, 2H, CH_2_), 3.381 - 3.440 (t, 2H, CH_2_), 5.203 - 5.311 (m, 4H, CH), 7.722 - 7.747 (t, 1H, NH); LC-MS (ESI) m/z: 323 (M+1), 321 (M-1); Anal. Calcd for C_20_H_38_N_2_O: C, 74.48; H, 11.88; N, 8.69; Found: C, 74.47; H, 11.86; N, 8.72.

### 3.3. Cytotoxicity Evaluation

#### 3.3.1. Cell Culture and Cell Viability Assay

The cytotoxic effects of DHA and LA derivatives against the breast cancer cell line (MCF-7) were assessed, with results compared to human dermal fibroblasts (HDFs) as a normal cell line. Both cell lines were provided by the Pasteur Institute of Iran (IPI). The cells were cultured in RPMI medium containing 10% fetal bovine serum (FBS) and 1% antibiotic solution (comprising 10,000 units of penicillin and 10 mg streptomycin in 0.9% NaCl). The cultures were incubated at 37°C with 5% CO_2_.

The MTT assay was used as a standard method for evaluating the inhibitory effect of compounds. This colorimetric assay is based on the conversion of yellow MTT (3-[4,5-dimethylthiazol-2-yl]-2,5-diphenyltetrazolium bromide) to purple formazan crystals via enzymatic reduction by NAD(P)H-dependent oxidoreductase enzymes in metabolically active cells (35). According to a previously reported method, after 24 hours of incubation of cells (15,000 cells per well), six different concentrations of D1-D8 (6.25 to 200 μM) and L1-L8 (3.125 to 100 μM) were added to the wells, with each concentration performed in triplicate. After 48 hours of incubation, the culture medium was completely removed from the wells. MTT solution, prepared at a concentration of 0.5 mg/mL in phosphate-buffered saline (PBS), was added (100 µL per well) and incubated for 4 hours to allow metabolically active cells to reduce the MTT to insoluble formazan crystals. Subsequently, 100 µL of solubilizing solvent containing 16% sodium dodecyl sulfate (SDS) was added to each well, followed by an additional 2 hours of incubation (36). Cell viability was determined using a Cell Imaging Multi-Mode Microplate Reader (Biotek, Cytation 3, USA), measuring absorbance at 570 nm (37, 38).

#### 3.3.2. Flow Cytometric Analysis

Apoptosis and necrosis were measured using a dead cell apoptosis kit with annexin V-FITC and propidium iodide (PI) (ApoFlowExFITC, Exbio, Czech Republic) according to the manufacturer’s protocol. The MCF-7 cells were cultured as described in the cell culture section (section 3.3.1). MCF-7 cells were seeded in six-well plates (2 × 10^5^ cells per well) and treated for 48 hours with synthesized compounds at three concentrations (their EC_50_, 2x EC_50_, ½x EC_50_) in triplicate. Cells were collected through trypsinization, rinsed twice with PBS, and then resuspended in 500 μL of binding buffer. The cell suspensions were subsequently incubated with 5 μL of annexin V-FITC and 5 μL of PI for 10 minutes at room temperature in a dark environment. The cells were then assessed immediately using flow cytometry, and the data were analyzed using FlowJo version 7.6.5 (Tree Star, Inc., Ashland, USA).

A distinguishing characteristic of cells undergoing apoptosis is the translocation of phosphatidylserine from the inner to the outer layers of the plasma membrane, which can be identified by annexin V in this assay. The PI identifies necrotic cells with compromised plasma membranes (39-44).

### 3.4. Molecular Modeling Study

Protein tyrosine phosphatase 1B was chosen as the receptor for docking studies of fatty acid derivatives (45, 46). The crystal structure of the PTP1B protein, PDB ID: 1Q1M (catalytic site) and 1T4J (allosteric domain), was downloaded from the protein data bank. The chemical structures of the designed fatty acid derivatives were drawn and optimized using the AMBER molecular mechanics method, employing the Polak-Ribiere algorithm via HyperChem 8.0 software. Preparation of ligands and protein was done with AutoDock Tools (Version 1.5.6rc3), which obtained PDBQT format, and molecular docking was carried out using AutoDock Vina (version 1.2.0) (47).

All water molecules and co-crystallized ligands were removed from the crystallographic structures, and all hydrogen atoms were added. Additionally, Kollman charges were applied to all ligands and proteins, and non-polar hydrogen atoms were merged. The center of each grid was adjusted to the coordinates of the center of the co-crystallized ligand. Docking was performed for both catalytic and allosteric sites. The grid dimensions were 30 × 30 × 30 points. Grid center coordinates were set at X = 56.74, Y = 31.37, Z = 23.07 for the allosteric site and X = 21.29, Y = 29.51, Z = 20.81 for the catalytic site to calculate the energetic map.

After 100 docking runs, the conformations with the best docking energy were analyzed using ViewerLite 5.0 and PyMOL software and were graphically displayed with Discovery Studio 4.5 Visualizer software. Validation was done with the PyMOL program (version 0.99rc6).

## 4. Results and Discussion

### 4.1. Chemistry

**Figure 2.**
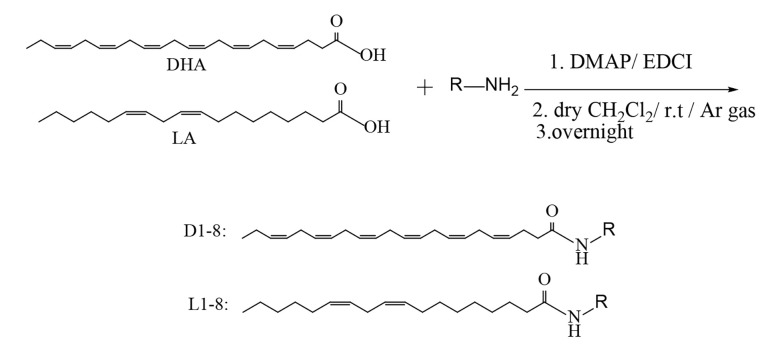
Procedure for the synthesis of compounds D1 - D7 and L1 - L7

**Figure 3.**
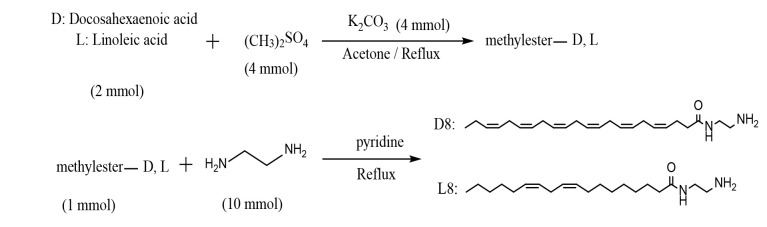
Procedure for the synthesis of compounds D8 and L8. D, docosahexaenoic acid (DHA); L, linoleic acid (LA)

In this study, sixteen derivatives of DHA and LA were designed and synthesized, as illustrated in Figures 2 and 3. The structures of compounds D1-D8 and L1-L8 were confirmed using ^1^H-NMR, LC-MS, FTIR, and elemental analysis. A summary of the synthesized amide derivatives is presented in Figure 1. 

Due to the high number of non-conjugated double bonds in DHA and the potential for side reactions involving these sites, all reactions were conducted under an inert nitrogen atmosphere to prevent unwanted oxidation or polymerization. Additionally, amide bonds are highly sensitive to hydrolysis in aqueous, acidic, and basic environments, necessitating the use of anhydrous solvents to improve reaction efficiency. The pH conditions were carefully optimized during workup and extraction to ensure maximum product stability and yield.

For purification, TLC was preferred over column chromatography due to the close movement of by-products to the target compounds, making plate chromatography a more effective separation technique. Since various monofunctional and bifunctional amines were employed in the synthesis, workup and purification methods were implemented accordingly for each compound.

It is noteworthy that for compounds D8 and L8, the lowest yields (approximately 60%) were obtained. This could be due to the bifunctionality of ethylenediamine, which increases the chances of side reactions occurring during the reaction course, leading to decreased yield. A possible strategy to avoid this issue would be to use a protected form of ethylenediamine, where only one amine group remains reactive during the reaction. Subsequent deprotection after the reaction could help improve the selectivity and overall yield of the desired amide products.

### 4.2. Docking Study

**Figure 4.**
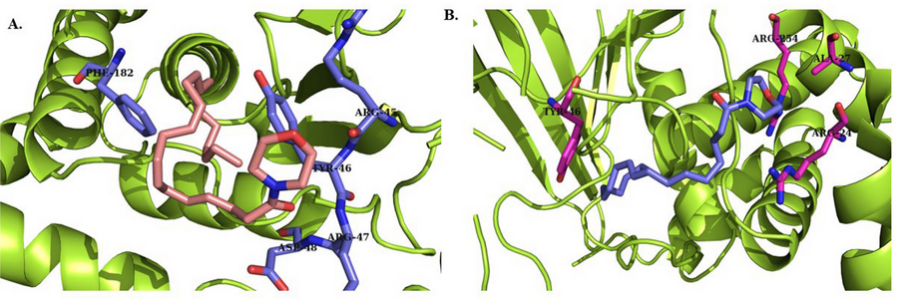
A, 3D image of the interaction of compound D3 with the catalytic site of PTP1B enzyme; B, 3D image of the interaction of compound L3 with the catalytic site of the enzyme

**Figure 5.**
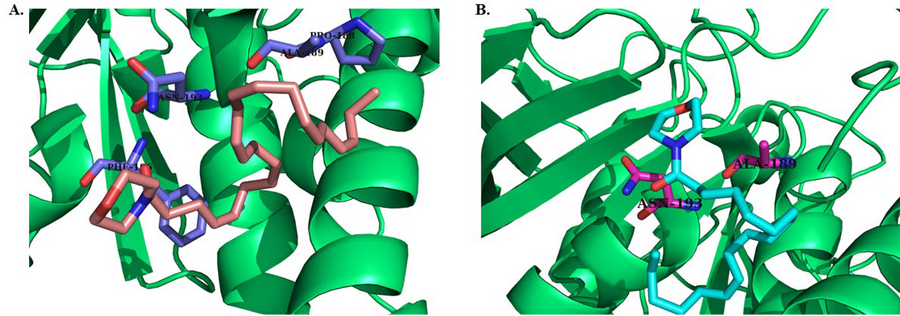
A, 3D image of the interaction of compound D3 with the allosteric site of PTP1B enzyme, B, 3D image of the interaction of compound L3 with the enzyme

The results of docking studies for two compounds (D3, L3) that showed more promising effects in biological evaluations are demonstrated in Figure 4 and 5. Compounds D3 and L3 exhibit satisfactory interaction with the active site of the PTP1B protein, with binding affinities of -6.0 and -5.3 kcal/mol, respectively, for the catalytic site, and -6.8 and -5.8 kcal/mol, respectively, for the allosteric site at a distance of less than 5 Å.

The important residues in the catalytic site include Arg 24 (distance: 2.61 Å), Ala 27 (distance: 4.03 Å), Arg 45 (distance: 3.43 Å), Tyr 46 (distance: 3.73 Å), Arg 47 (distance: 2.68 Å), Asn 48 (distance: 2.21 Å), Phe 182 (distance: 4.93 Å), and Arg 254 (distance: 2.54 Å) (Figure 4). For the allosteric site, the key residues are Pro 188 (distance: 4.04 Å), Ala 189 (distance: 3.89 Å), Asn 193 (distance: 3.16 Å), and Phe 196 (distance: 3.62 Å) (Figure 5). 

In particular, these two compounds interact with residues responsible for the potency and selectivity of the active site. The best conformation obtained from docking of co-crystallized ligands superimposed with the co-crystallized ligands in 1Q1M as the catalytic site and 1T4G as the allosteric site using PyMOL software confirms that compounds D3 and L3 are correctly located in the catalytic and allosteric sites. The root mean square deviation (RMSD) results were 0.4 and 1.2 Å for the catalytic and allosteric sites, respectively. Since the obtained RMSD values were less than 2 Å, the docking method was considered valid.

To the best of our knowledge, docking analysis of DHA and LA derivatives with PTP1B has not been reported, except for a blind docking of compound DHA with the PTP1B enzyme conducted by Kuban-Jankowska et al. (26). They assessed the binding conformations and potential interactions of DHA with various binding sites of the enzyme, reporting probable interactions of DHA with Lys116, Tyr46, and Arg221 at the allosteric site, and possible interactions in the second binding site with Arg79 and Asp236 (26).

### 4.3. Biological Evaluations

#### 4.3.1. Functional Fatty Acid Derivatives Inhibited the Growth of MCF-7 Cells In Vitro

**Figure 6.**
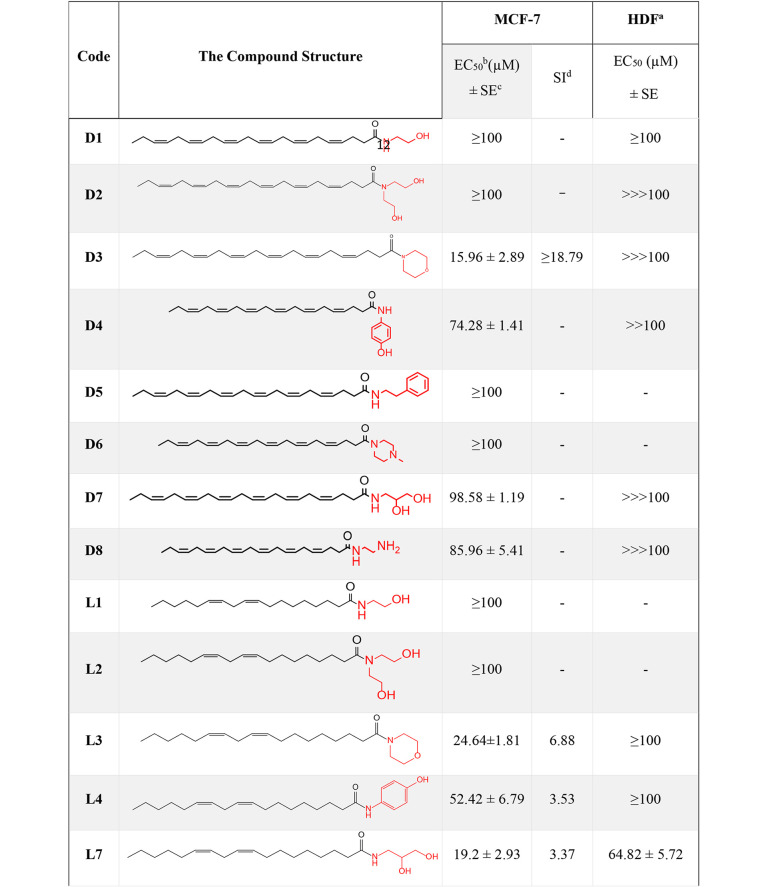
The EC_50_ of synthesized DHA (D1 - D8) and LA (L1 - L8) derivatives in MCF-7 and human dermal fibroblasts (HDFs)

A series of amide derivatives of DHA and LA were synthesized and evaluated for their cytotoxic effects on MCF-7 breast cancer cells and HDF normal cells. As illustrated in Figure 6, the cytotoxicity results revealed that compounds D3, L7, and L3 demonstrated the most promising cytotoxic activity, with EC_50_ values of 15.96 ± 2.89 µM, 19.2 ± 2.93 µM, and 24.64 ± 1.81 µM, respectively.

To assess selectivity, the Selectivity Index (SI) was calculated as the ratio of EC_50_ against fibroblasts to EC_50_ against MCF-7 cells, with D3 (SI = 18.79), L3 (SI = 6.88), and L7 (SI = 3.37) showing superior selectivity compared to cisplatin (SI = 1.18, EC_50_ = 0.85 µM). The significant selectivity of DHA derivatives can be attributed to the fundamental biological differences between cancer and normal cells, which make cancer cells more susceptible to their effects while sparing normal fibroblasts.

Several mechanisms contribute to this preferential cytotoxicity:

(1) Induction of oxidative stress: Cancer cells naturally exhibit high oxidative stress, making them more vulnerable to further increases in reactive oxygen species (ROS) caused by DHA oxidation (27). The peroxidation of PUFAs leads to the generation of cytotoxic lipid peroxides, which induce apoptosis in cancer cells, whereas normal cells, with more efficient antioxidant defense systems, can better tolerate this oxidative burden (9, 10, 48, 49).

(2) Disruption of lipid rafts and oncogenic signaling: Docosahexaenoic acid and EPA derivatives readily incorporate into cellular membranes, altering membrane lipid composition and destabilizing lipid rafts, which are critical for oncogenic signaling pathways. By interfering with lipid raft domains, these compounds inhibit receptor tyrosine kinases (RTKs), PI3K/Akt, and MAPK pathways, which are hyperactivated in cancer cells to promote survival and proliferation (50, 51). Normal cells, which do not rely on these altered signaling pathways for survival, remain relatively unaffected.

(3) Preferential uptake by cancer cells: Cancer cells exhibit increased expression of fatty acid transporters such as CD36 and FATP, which enhances the selective accumulation of DHA and EPA derivatives in tumor cells. This leads to a higher intracellular concentration of lipid peroxides and apoptotic signals, selectively affecting cancer cells while sparing fibroblasts, which have lower uptake of PUFAs (52-54).

(4) Activation of apoptotic pathways: Docosahexaenoic acid and EPA derivatives enhance mitochondrial membrane permeability, leading to cytochrome c release and caspase-dependent apoptosis. Cancer cells, which often have dysregulated apoptotic pathways, are more susceptible to these disruptions, whereas normal cells with functional mitochondrial integrity can better regulate apoptosis and survive (13).

While cisplatin displayed the highest potency (EC_50_ = 0.85 µM), its low SI (1.18) indicates a significant lack of selectivity, meaning it is equally toxic to normal fibroblasts and cancer cells. In contrast, D3, L3, and L7 demonstrated higher selectivity, suggesting a potentially better therapeutic window with reduced off-target toxicity. The severe side effects of cisplatin, such as nephrotoxicity, neurotoxicity, and myelosuppression, often limit its long-term clinical use (55). In comparison, DHA-based compounds have been reported to exhibit lower systemic toxicity, making them promising candidates for further development as safer anti-cancer agents.

Comparing the cytotoxic effect of DHA on MCF-7 breast cancer cell viability using the MTT assay, it was revealed that DHA could reduce cell viability in a concentration-dependent manner. Cell viability was significantly reduced at 150 μM DHA (26), whereas in our study, derivatives of DA and even LA were able to reduce cancer cell viability with lower EC_50_.

#### 4.3.2. Evaluation of Apoptosis Using Flow Cytometry

**Figure 7.**
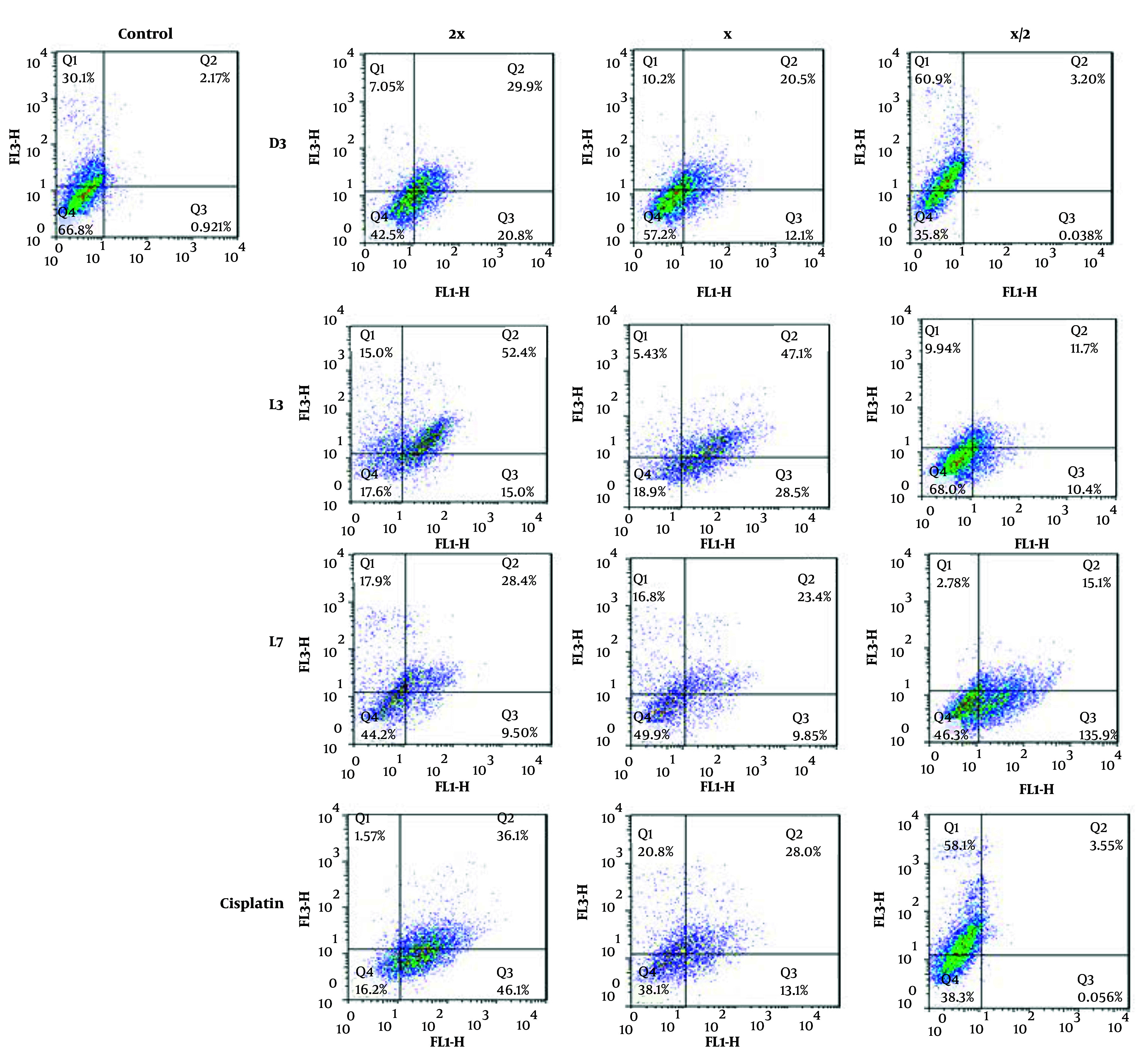
The effect of compounds D3, L3, and L7 on early and late apoptosis or necrosis in MCF-7 cells detected by flow cytometry. Diagrams of annexin V- FITC/PI staining of MCF-7 cells were presented. The quadrants were as follows: Q1, annexin V/PI+, necrosis; Q2, annexin V+/PI+, late or secondary apoptosis; Q3, annexin V/PI, early apoptosis; Q4, annexin V+/PI, normal cells without apoptosis or necrosis.

**Figure 8.**
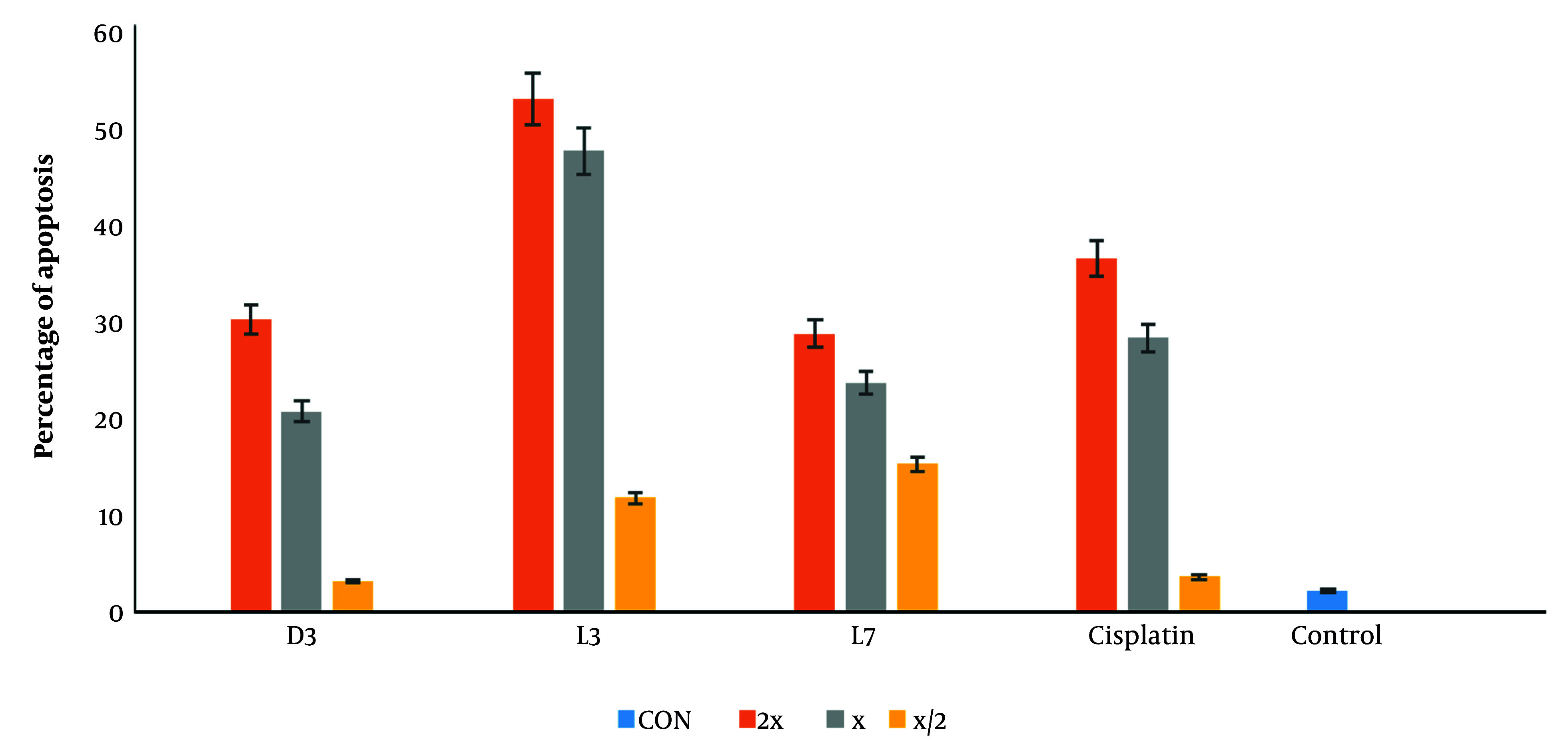
Analysis of the cell apoptosis with annexin-V/PI staining, using flow cytometry, the groups treated with fatty acid derivatives at (EC_50_ = x, 2x, x/2) µM concentration for 48 h. Data are presented as mean ± SD (n = 3 replicate).

To further investigate the mode of cell death, flow cytometry was performed on MCF-7 cells treated with D3, L3, and L7, using annexin V-FITC/PI staining (Figure 7). The analysis confirmed that these derivatives predominantly induced apoptosis rather than necrosis, reinforcing the selective cytotoxicity observed in the MTT assay. With increasing concentrations (½x, x, 2x EC_50_), apoptotic cell death progressively increased, with D3 and L3 exhibiting the highest apoptotic potential (Figure 8). 

The results suggest that these derivatives may activate both early and late apoptosis in MCF-7 cells, further validating the pro-apoptotic effects of DHA and EPA-based compounds. Previous studies have demonstrated that DHA exerts its anti-cancer effects through apoptosis induction (13, 27, 56), mitochondrial dysfunction, and oxidative stress (9, 10, 49). Our findings confirm that amide derivatives of DHA and EPA follow a similar mechanism, reinforcing their potential as selective anti-cancer agents.

## 5. Conclusions

This study highlights the therapeutic potential of amide derivatives of DHA and EPA as selective anti-cancer agents. The molecular modeling and docking studies demonstrated that the designed compounds can orient properly in the active site of PTP1B and are capable of forming hydrogen bonds and hydrophobic interactions with amino acids.

Among the tested compounds, D3 demonstrated the highest SI (18.79), followed by L3 (6.88) and L7 (3.37), making them promising candidates for further preclinical evaluation. Unlike cisplatin, which exhibits poor selectivity and significant toxicity, DHA-based derivatives selectively target cancer cells through multiple mechanisms, including oxidative stress induction, lipid raft disruption, enhanced uptake, and apoptosis activation. In our study, D3, L3, and L7 were selected for further studies on their mechanism of action.

Finally, compounds D3 and L3, with cytotoxic activities showing EC_50_s of 15.96 ± 2.89 µM and 24.64 ± 1.81 µM and apoptosis percentages of 20.5% and 47.1%, respectively, were recognized as the most potent anti-cancer compounds compared to the rest of the derivatives. Although these compounds exhibit lower potency than cisplatin, their significantly higher SI suggests a safer profile, which could be advantageous in reducing side effects.

Future studies should focus on mechanistic assays, in vivo evaluations, and structure-activity relationship (SAR) studies to optimize their anti-cancer potential.

ijpr-24-1-159523-s001.pdf

## Data Availability

The data presented in this study are uploaded during submission as a supplementary file.
