# Supplementary material for: Design, Synthesis, and Evaluation of Cytotoxic Effects of Functional Fatty Acid Derivatives as Potential Antineoplastic Agents for Breast Cancer
Source: Iran J Pharm Res. 2025 Mar 15;24(1):e159523. doi: 10.5812/ijpr-159523 (PMC12297039; doi:10.5812/ijpr-159523)
Supplement: ijpr-24-1-159523-s001.pdf [file ijpr-24-1-159523-s001.pdf]

(4Z,7Z,10Z,13Z,16Z,19Z)-N-(2-hydroxyethyl) docosa-4,7,10,13,16,19-hexaenamide (D1)

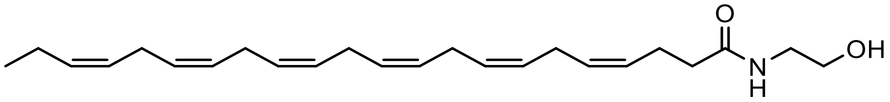

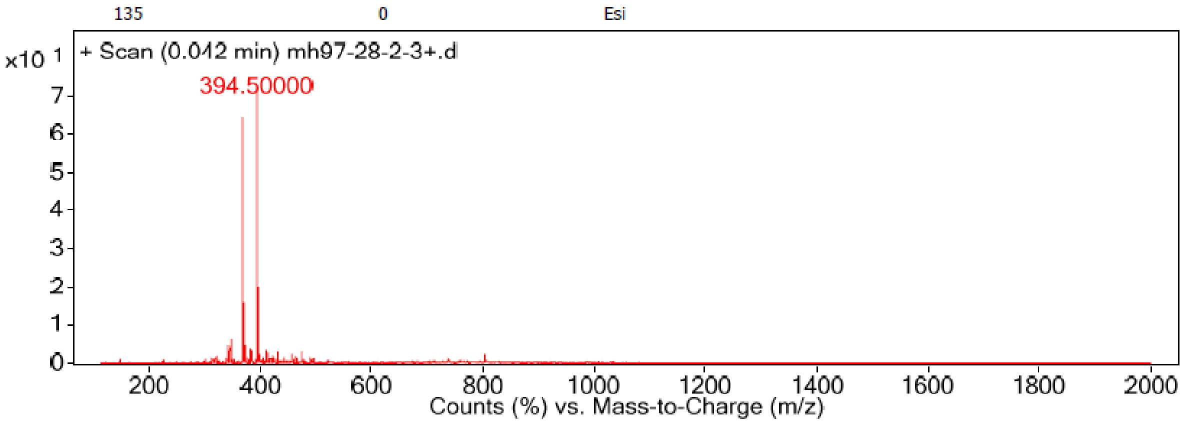

Figure 1. ESI-MS spectrum of compound D1

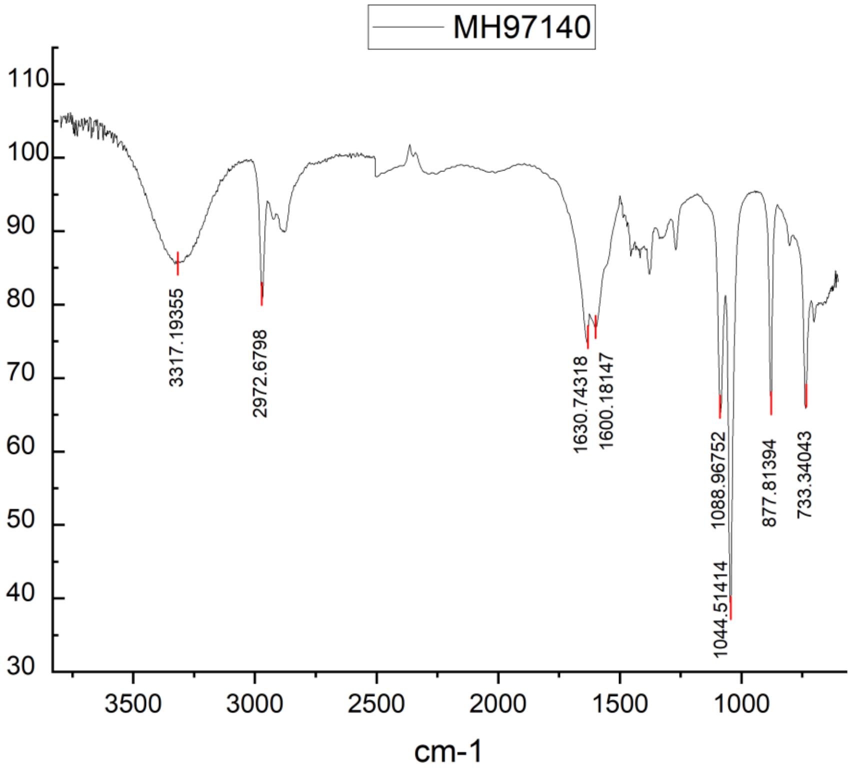

Figure 2. FT-IR spectrum of compound D1

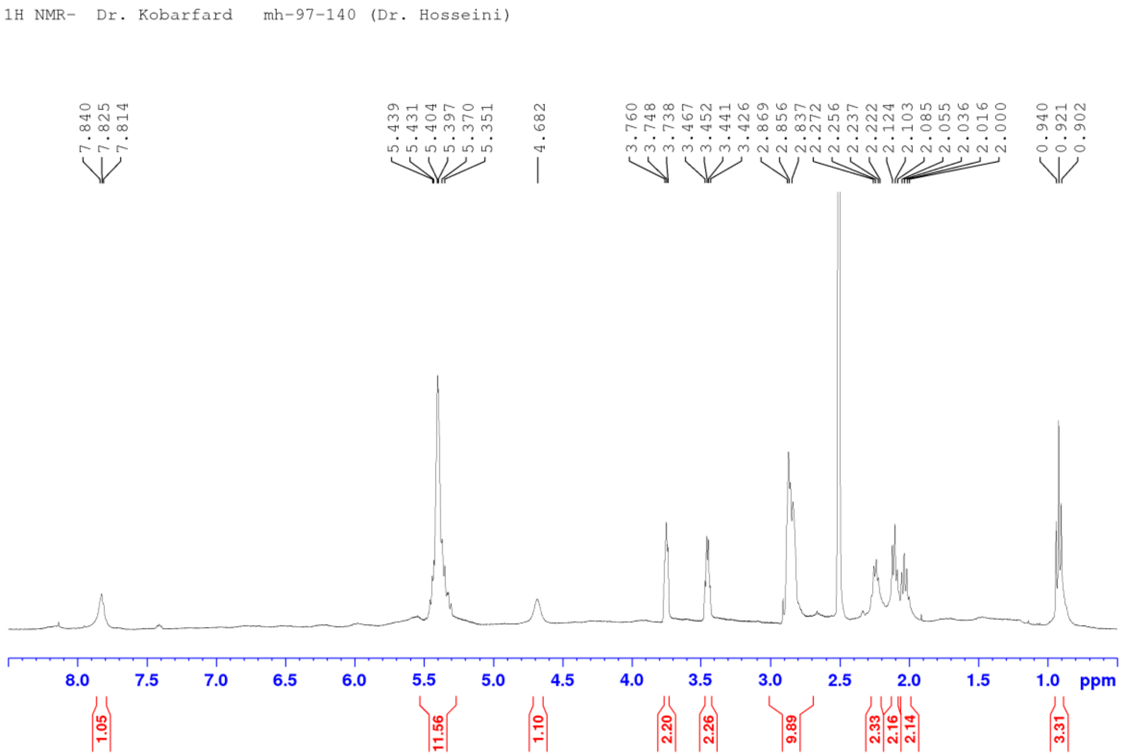

Figure 3. ^{1}H-NMR spectrum of compound D1

(4Z,7Z,10Z,13Z,16Z,19Z)-N,N-bis(2-hydroxyethyl)docosa-4,7,10,13,16,19-hexaenamide (D2)

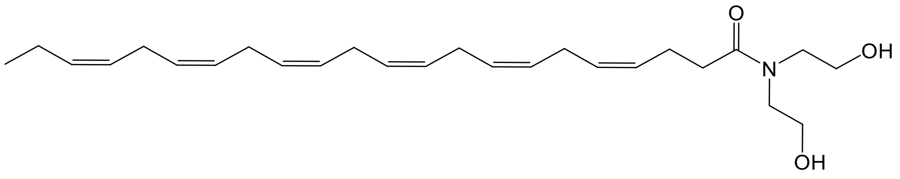

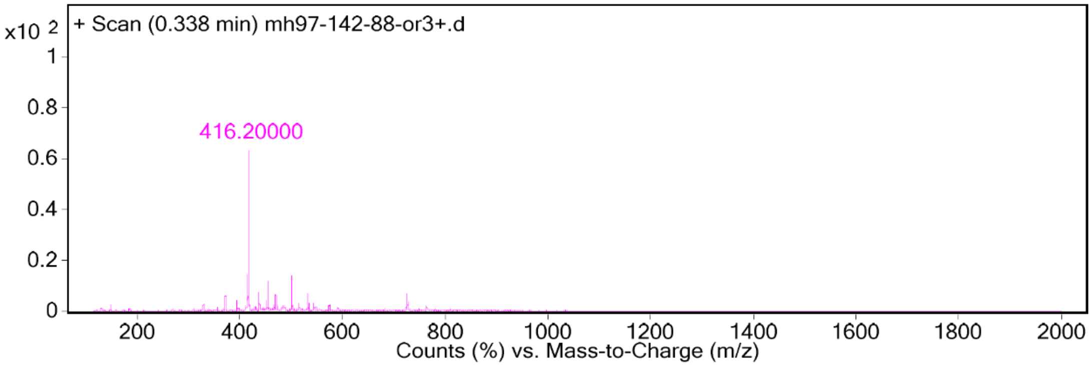

Figure 4. ESI-MS spectrum of compound D2

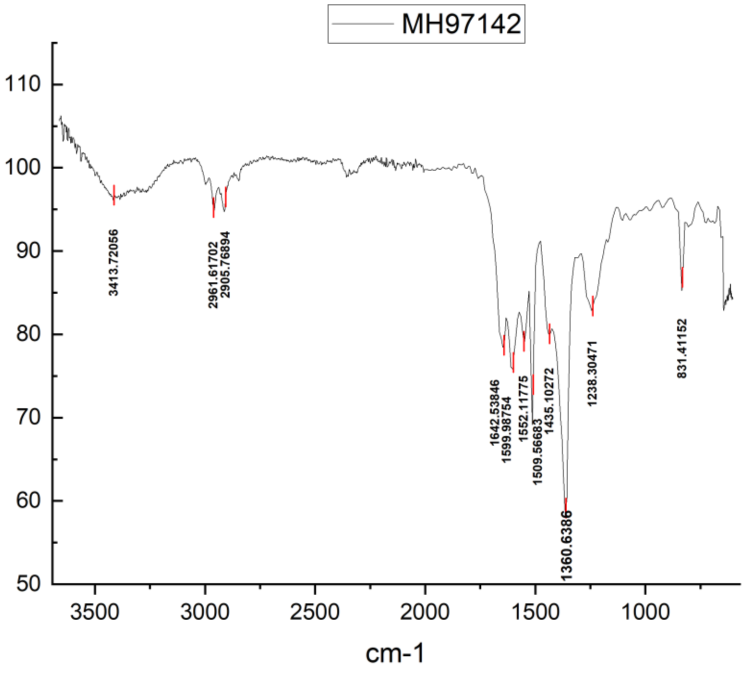

Figure 5. FT-IR spectrum of compound D2

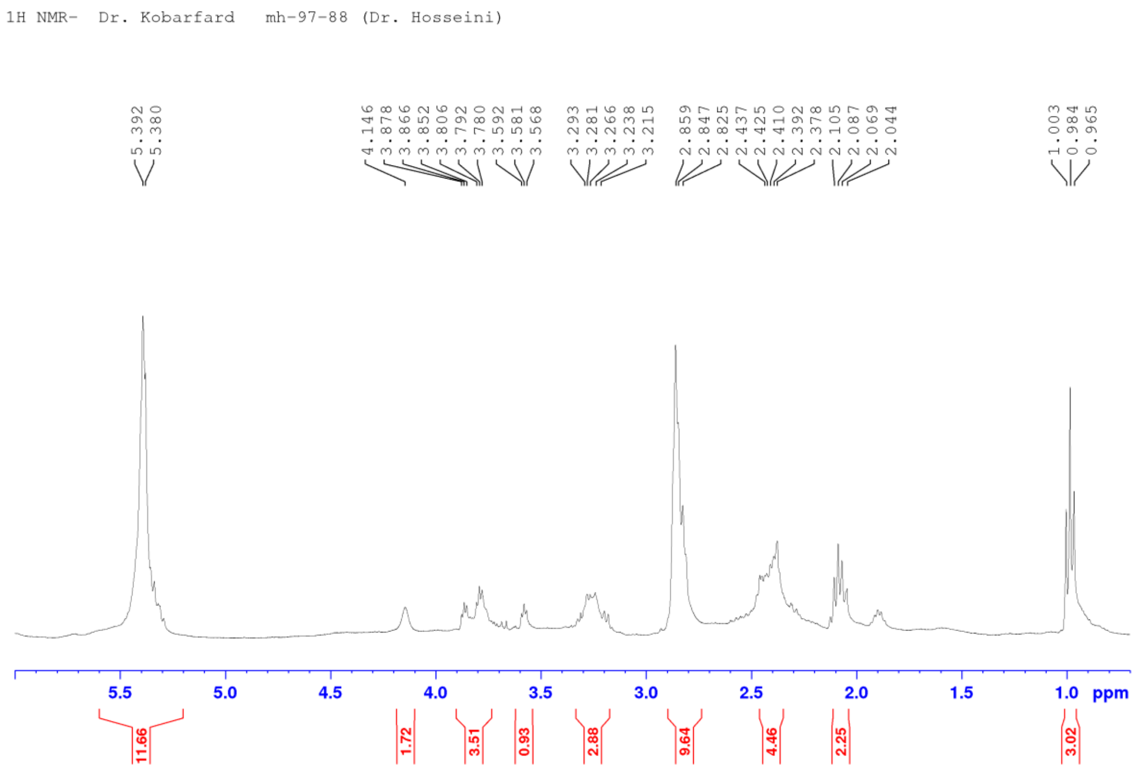

Figure 6. ^{1}H-NMR spectrum of compound D2

4-((4Z,7Z,10Z,13Z,16Z,19Z)-docosa-4,7,10,13,16,19-hexaen-1-yl)morpholine (D3)

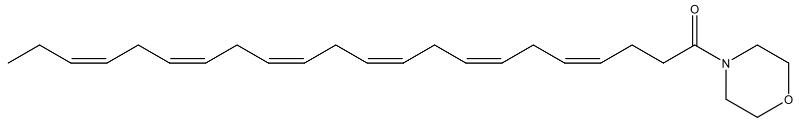

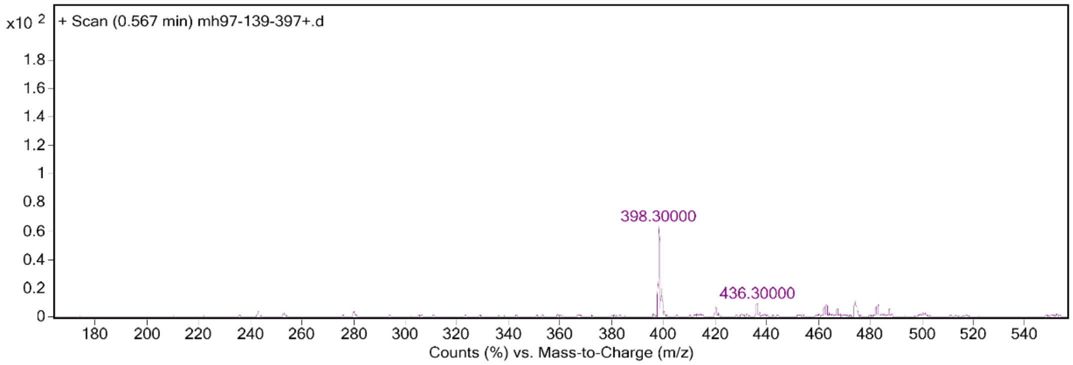

Figure 7. ESI-MS spectrum of compound D3

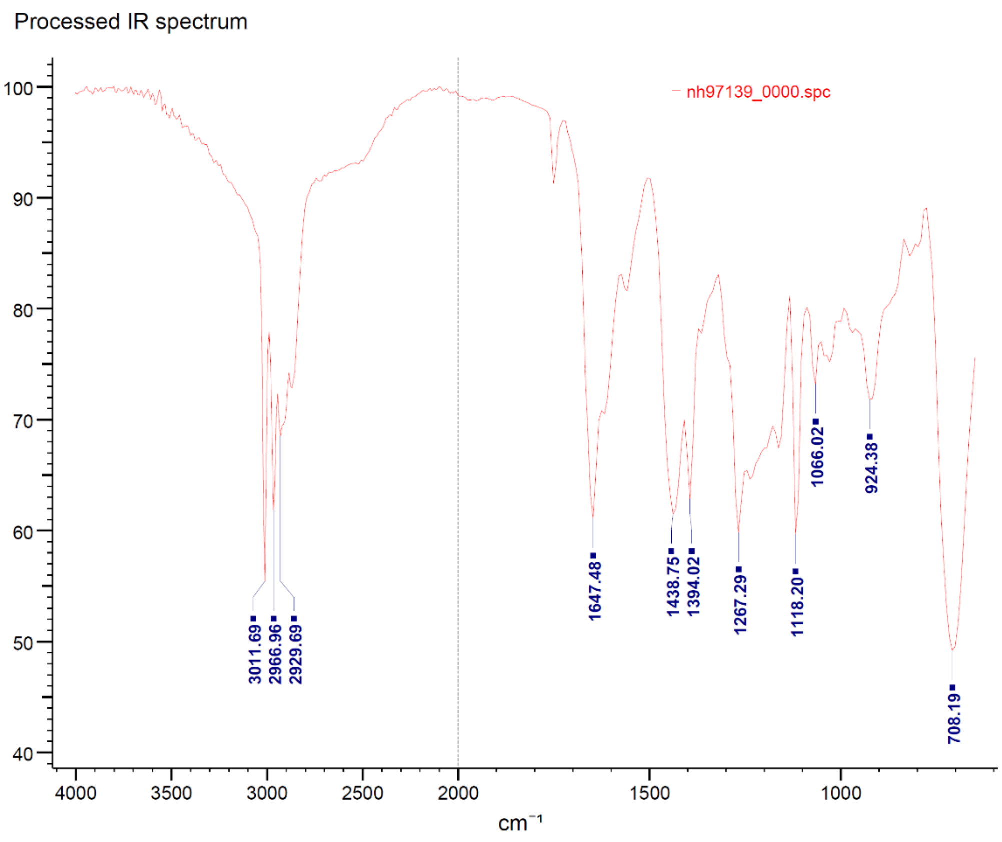

Figure 8. FT-IR spectrum of compound D3

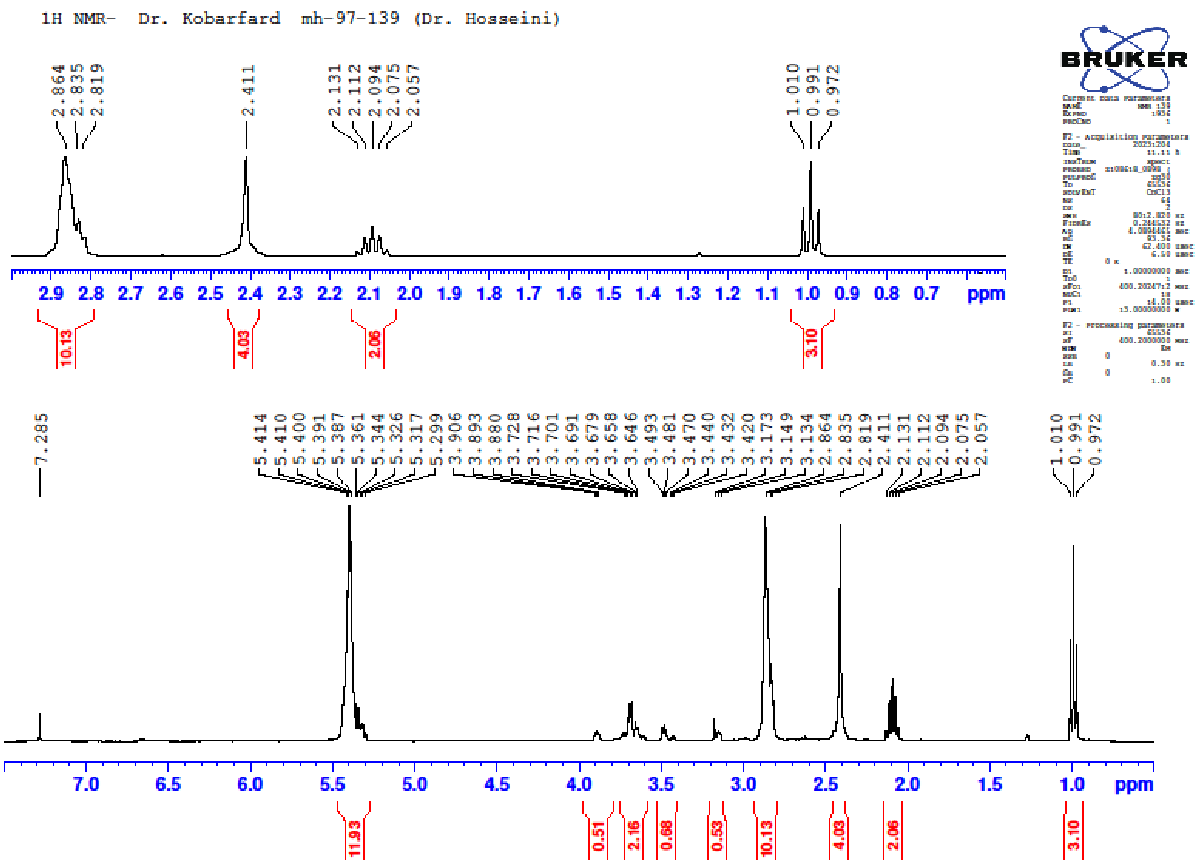

Figure 9. ^{1}H-NMR spectra of compound D3

(4Z,7Z,10Z,13Z,16Z,19Z)-N-(4-hydroxyphenyl)docosa-4,7,10,13,16,19-hexaenamide (D4)

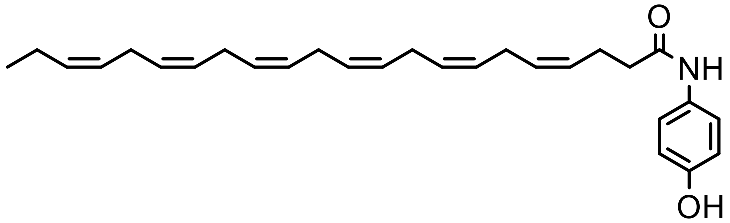

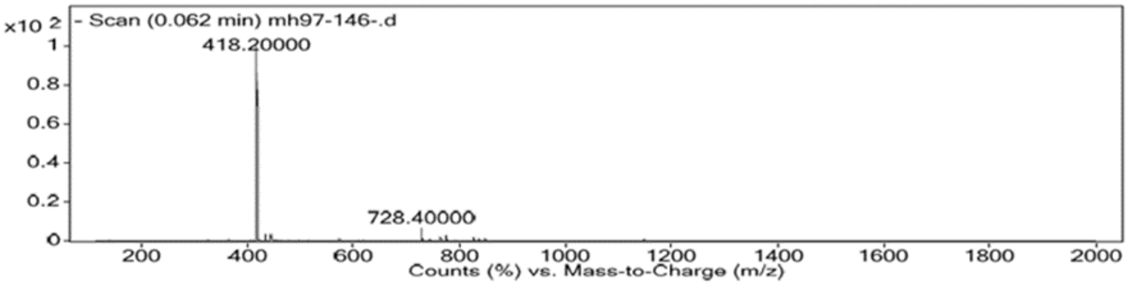

Figure 10. ESI-MS spectrum of compound D4

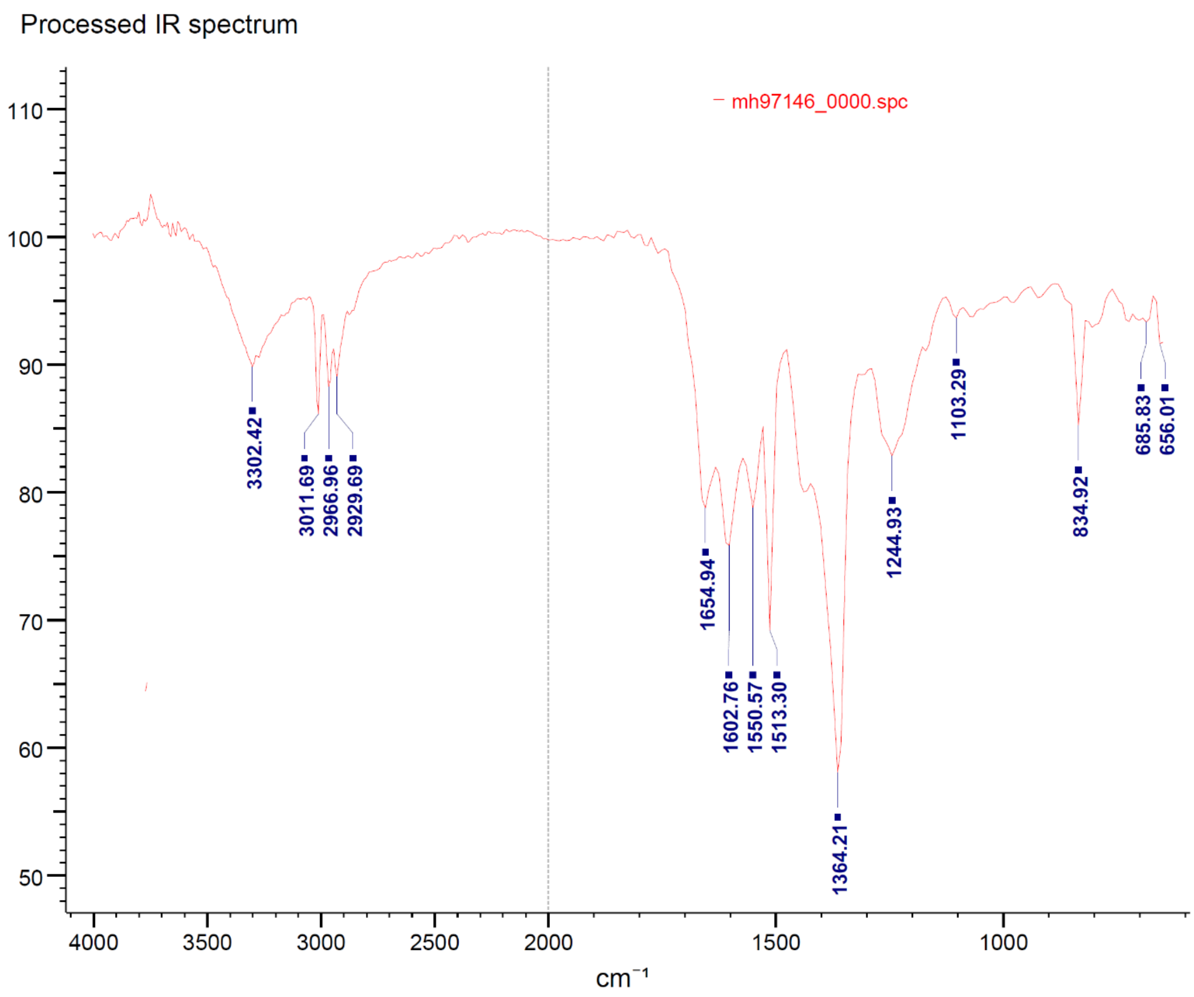

Figure 11. FT-IR spectrum of compound D4

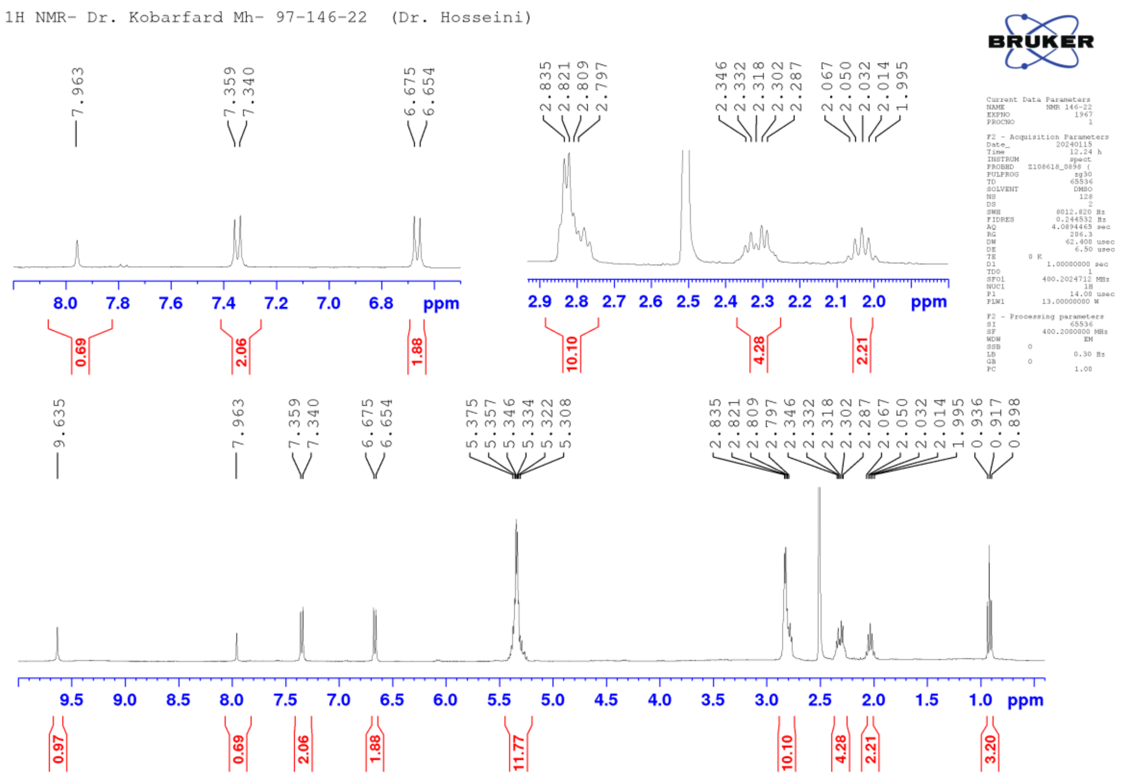

Figure 12.^{1}H-NMR spectra of compound D4

(4Z,7Z,10Z,13Z,16Z,19Z)-N-phenethyldocosa-4,7,10,13,16,19-hexaenamide (D5)

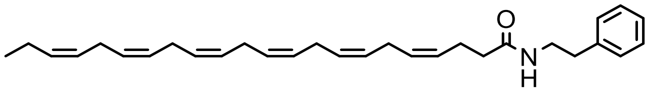

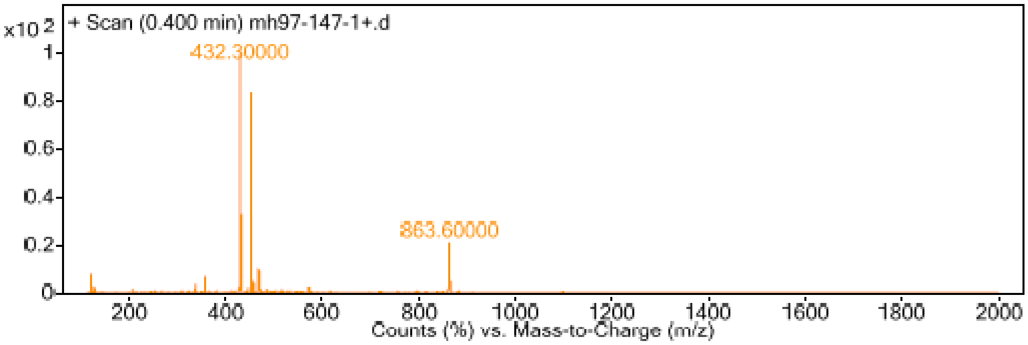

Figure 13. ESI-MS spectrum of compound D5

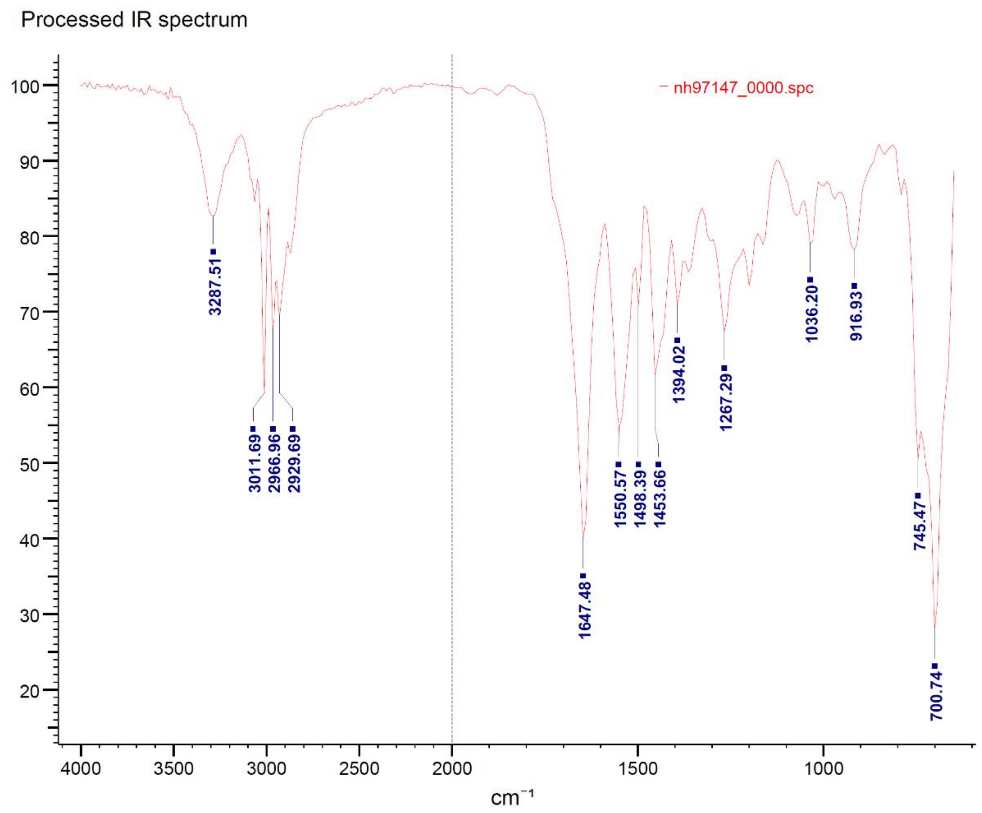

Figure 14. FT-IR spectrum of compound D5

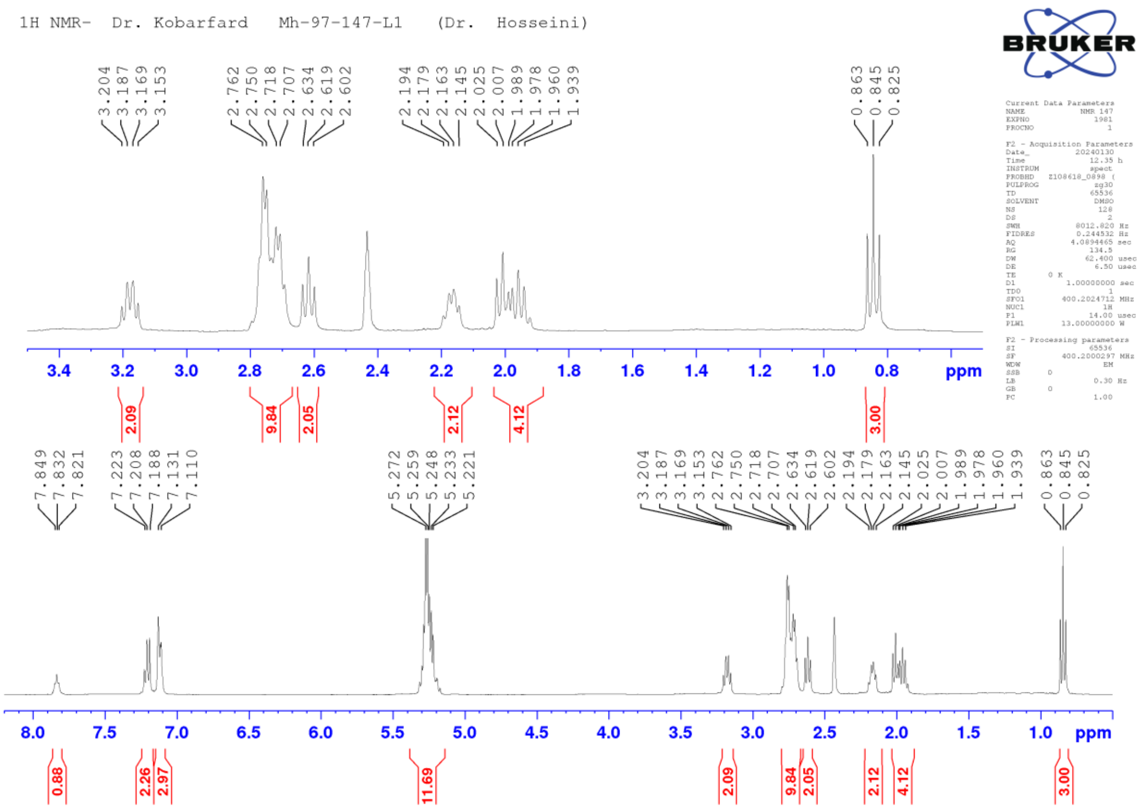

Figure 15. ^{1}H-NMR spectra of compound D5

(4Z,7Z,10Z,13Z,16Z,19Z)-1-(4-methylpiperazin-1-yl)docosa-4,7,10,13,16,19-hexaen-1-one (D6)

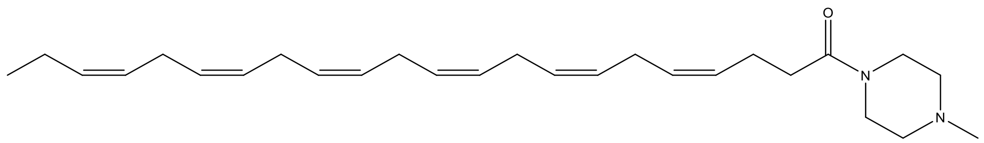

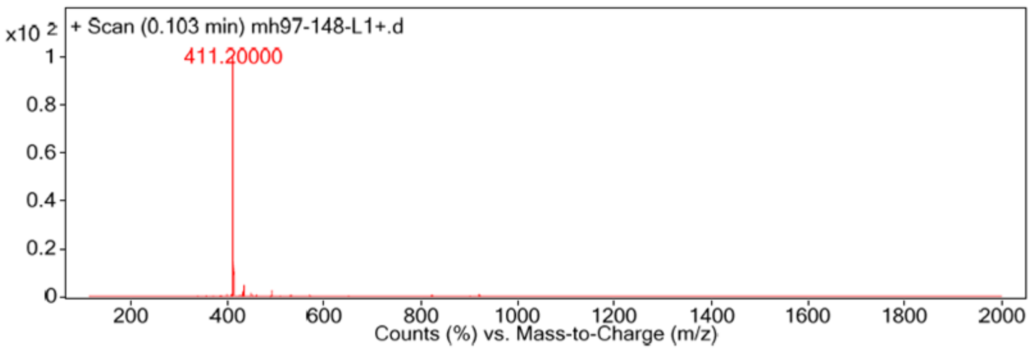

Figure 16. ESI-MS spectrum of compound D6

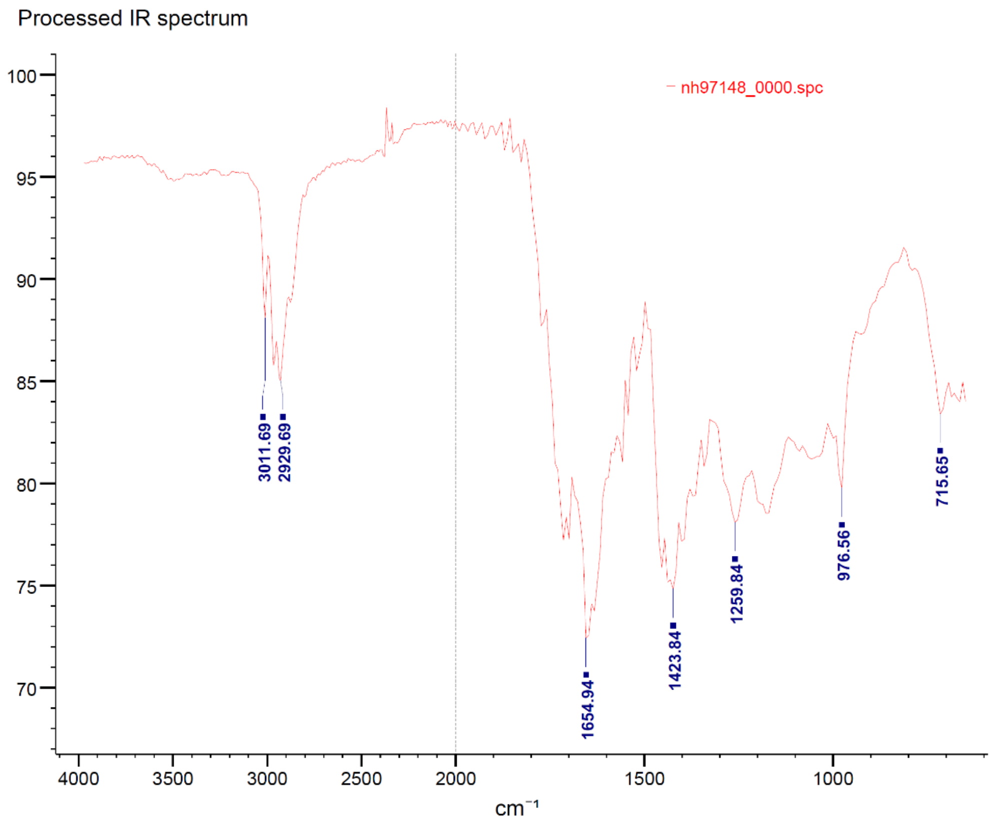

Figure 17. FT-IR spectrum of compound D6

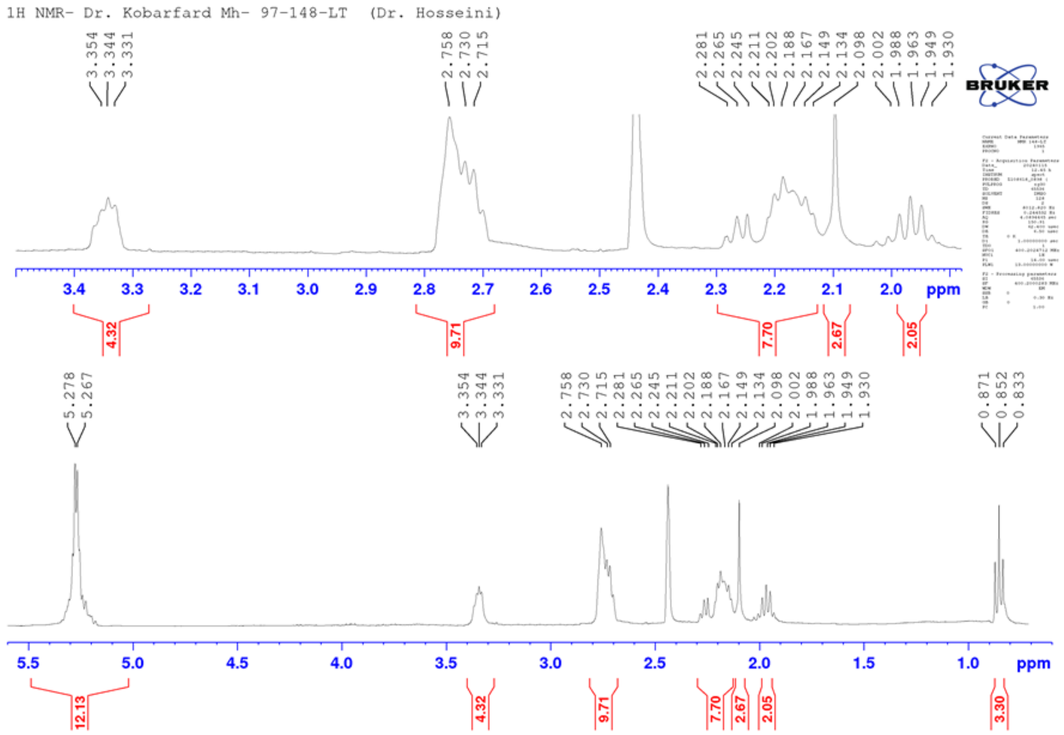

Figure 18. ^{1}H-NMR spectra of compound D6

(4Z,7Z,10Z,13Z,16Z,19Z)-N-(2,3-dihydroxypropyl)docosa-4,7,10,13,16,19-hexaenamide (D7)

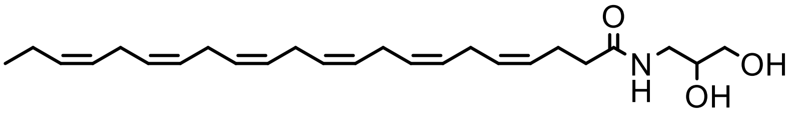

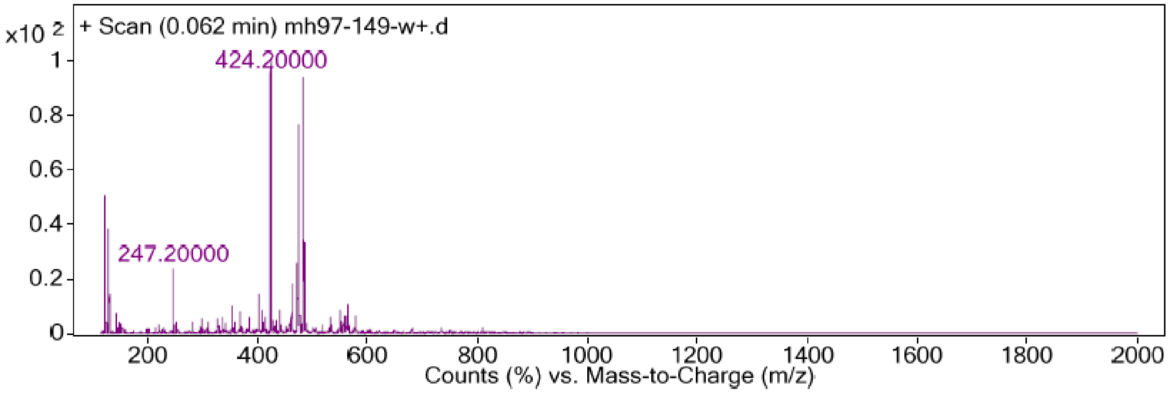

Figure 19. ESI-MS spectrum of compound D7

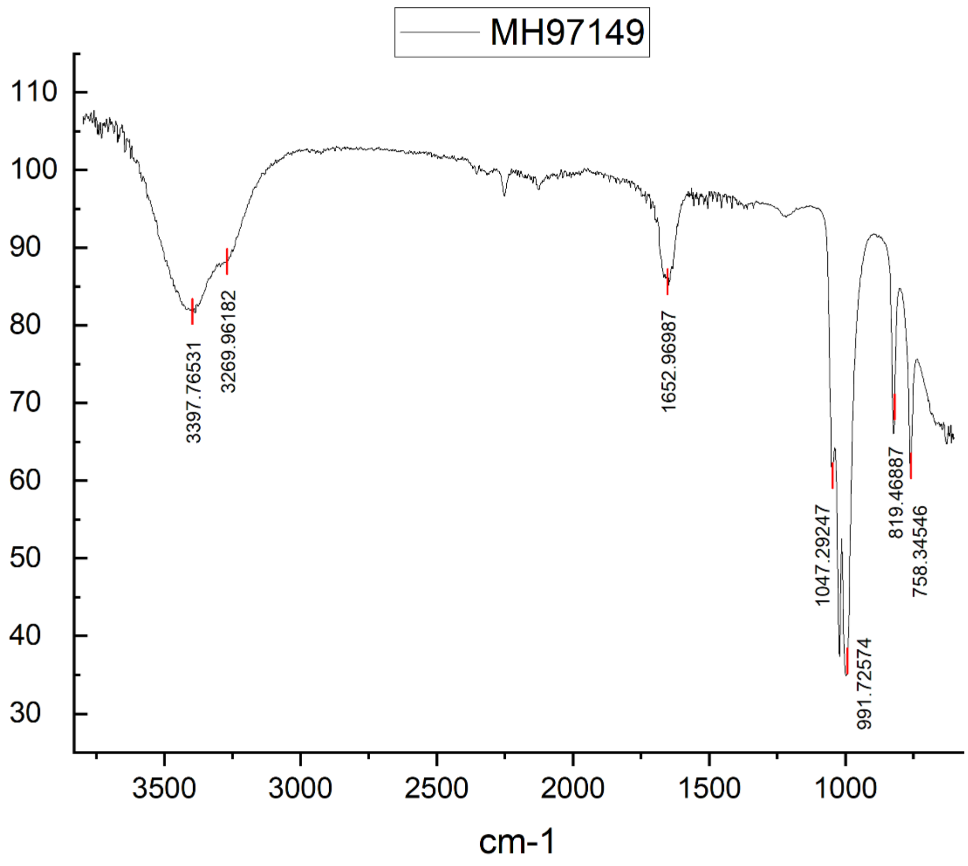

Figure 20. FT-IR spectrum of compound D7

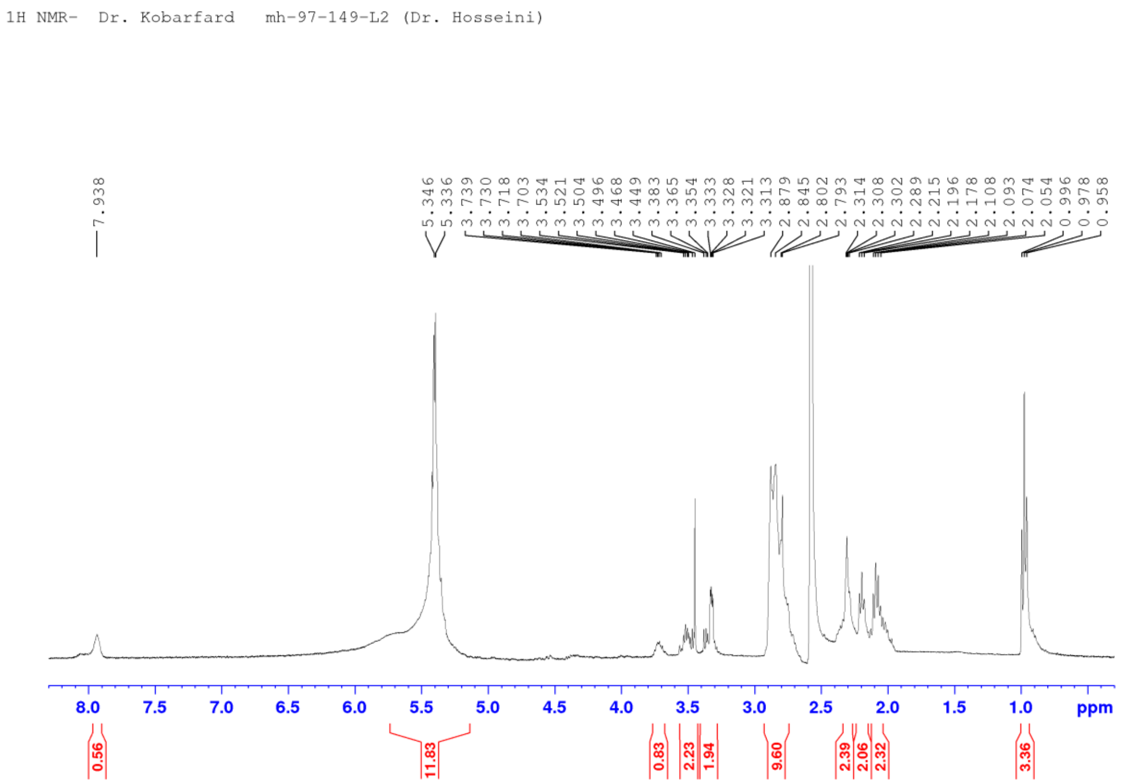

Figure 21.^{1}H-NMR spectrum of compound D7

(4Z,7Z,10Z,13Z,16Z,19Z)-N-(2-aminoethyl)docosa-4,7,10,13,16,19-hexaenamide (D8)

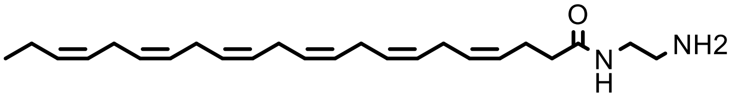

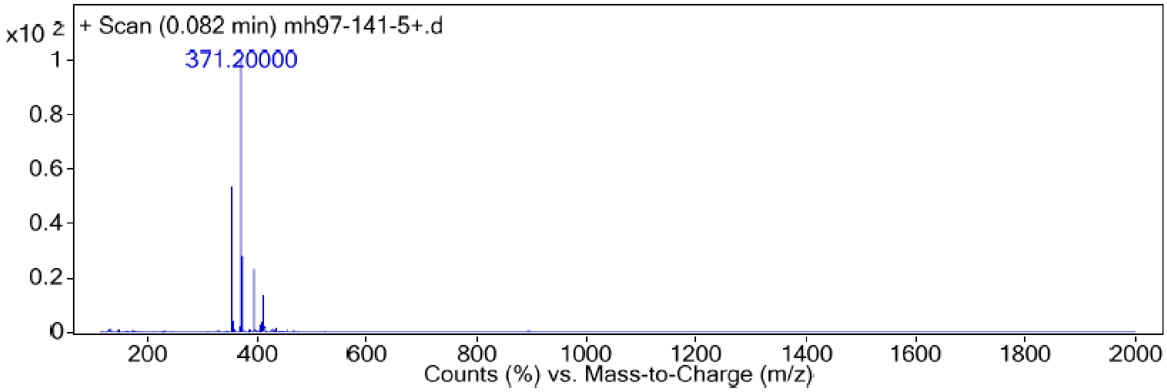

Figure 22. ESI-MS spectrum of compound D8

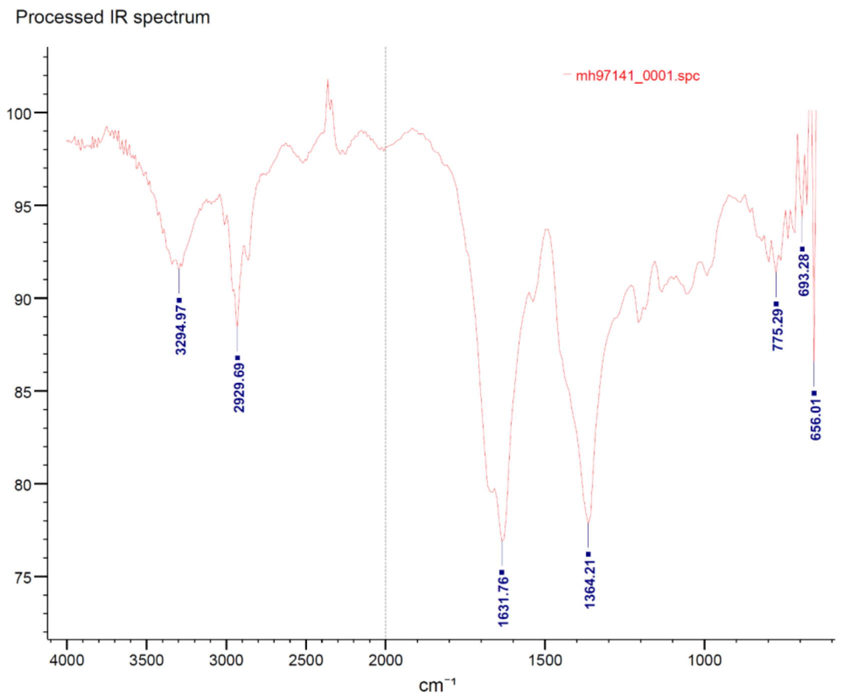

Figure 23. FT-IR spectrum of compound D8

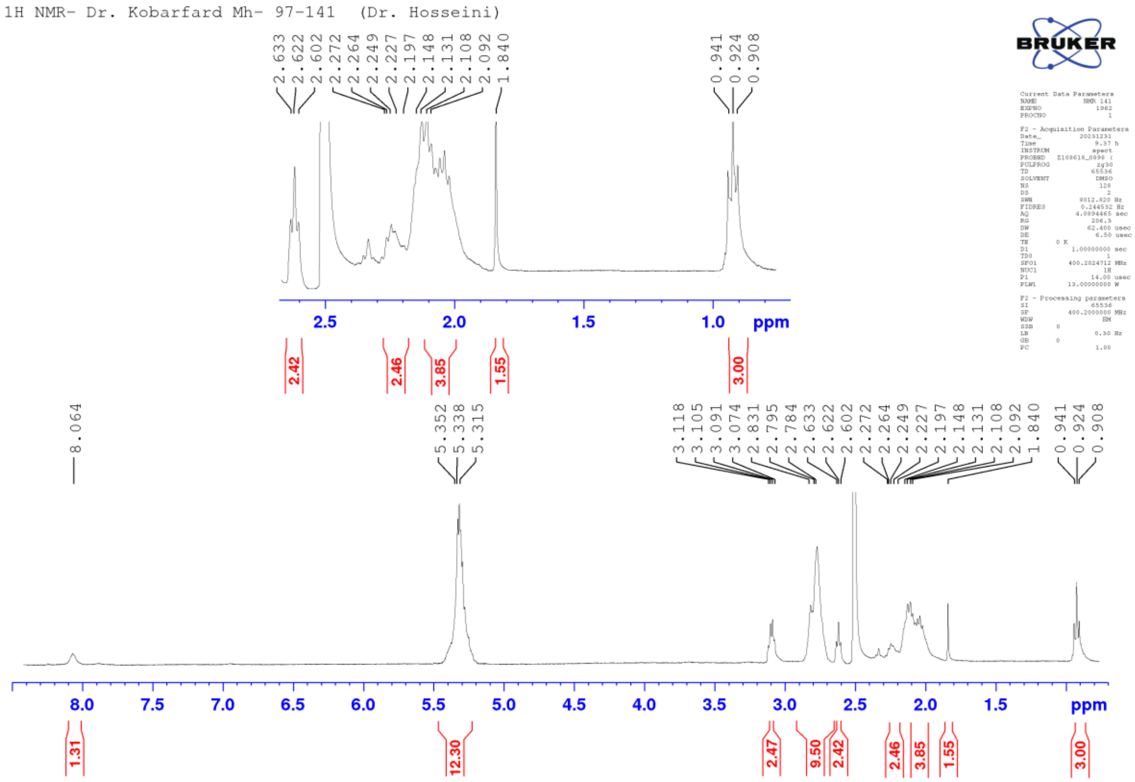

Figure 24. ^{1}H-NMR spectra of compound D8

(9Z,12Z)-N-(2-hydroxyethyl)octadeca-9,12-dienamide (L1)

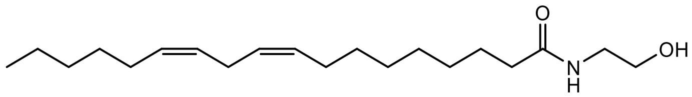

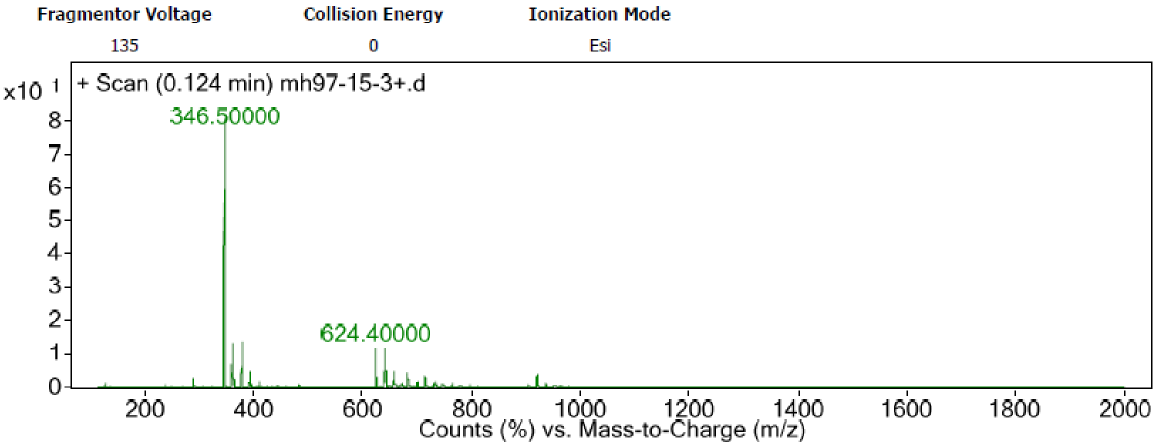

Figure 25. ESI-MS spectrum of compound L1

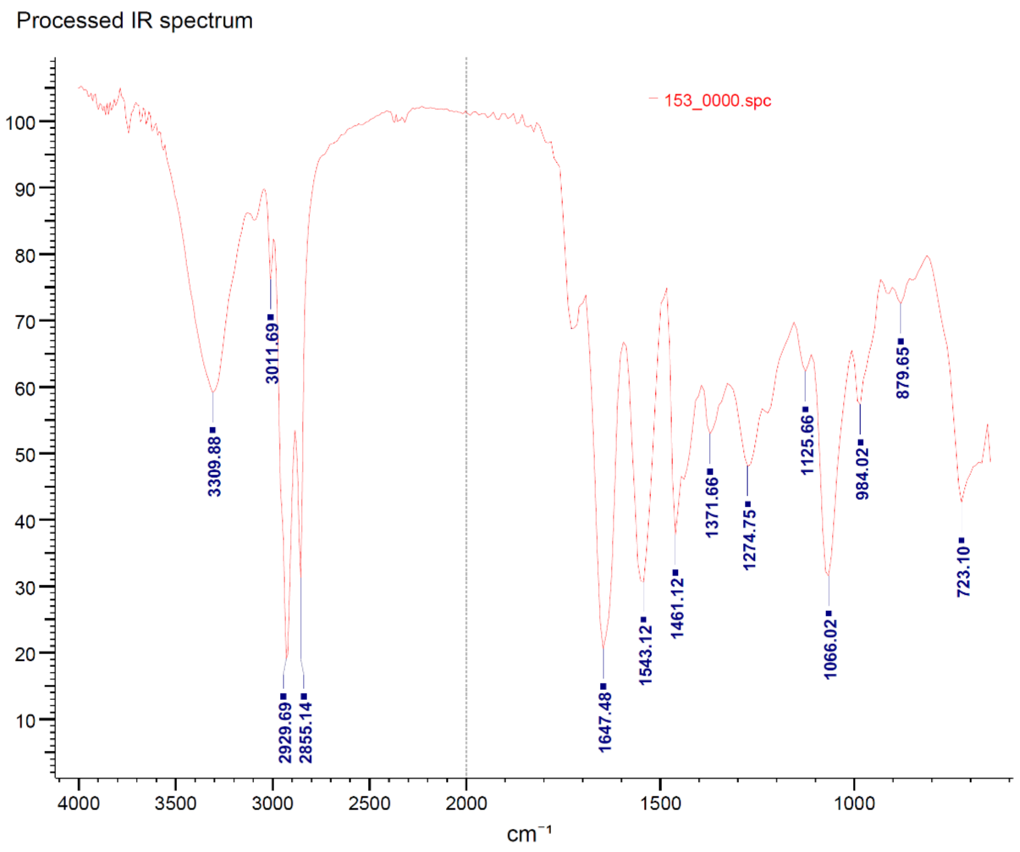

Figure 26. FT-IR spectrum of compound L1

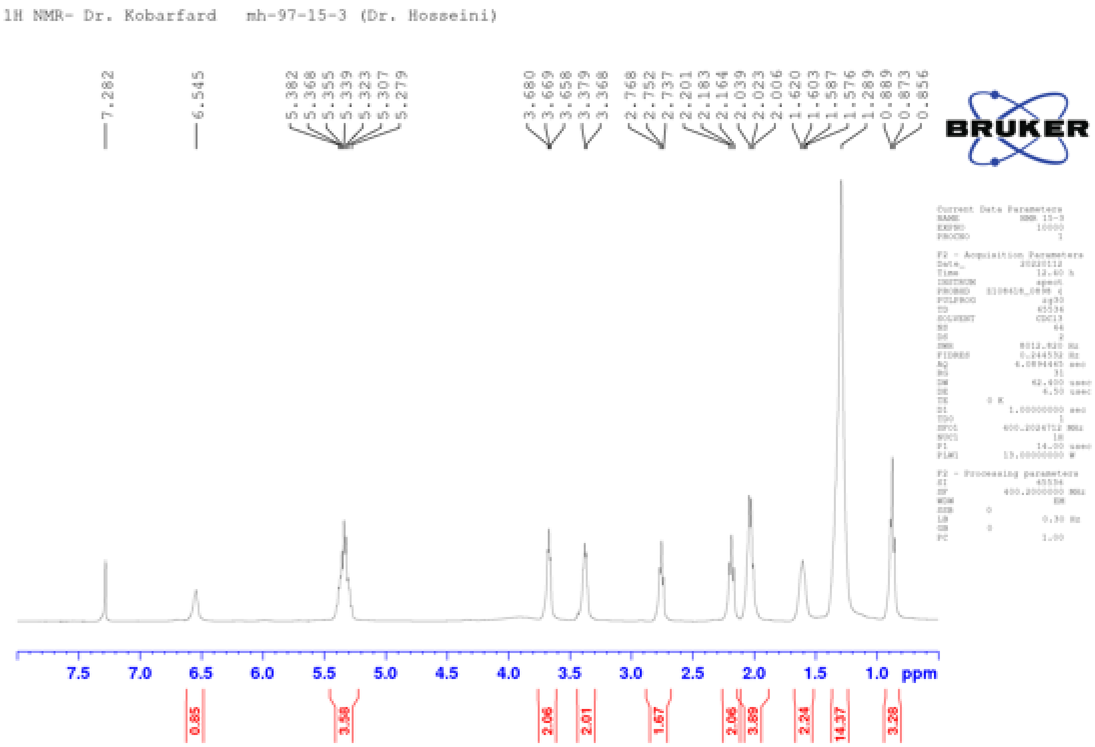

Figure 27. ^{1}H-NMR spectra of compound L1

(14-2,L2): (9Z,12Z)-N,N-bis(2-hydroxyethyl)octadeca-9,12-dienamide (L2)

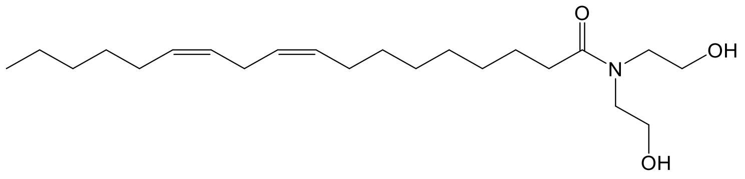

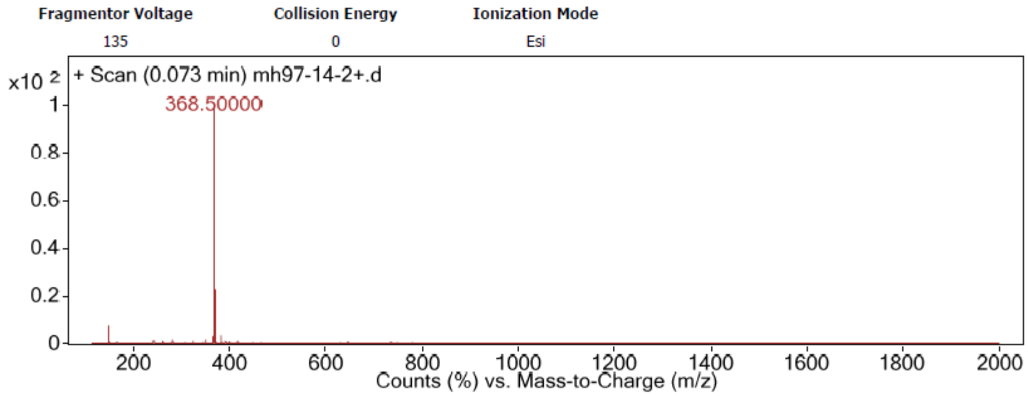

Figure 28. ESI-MS spectrum of compound L2

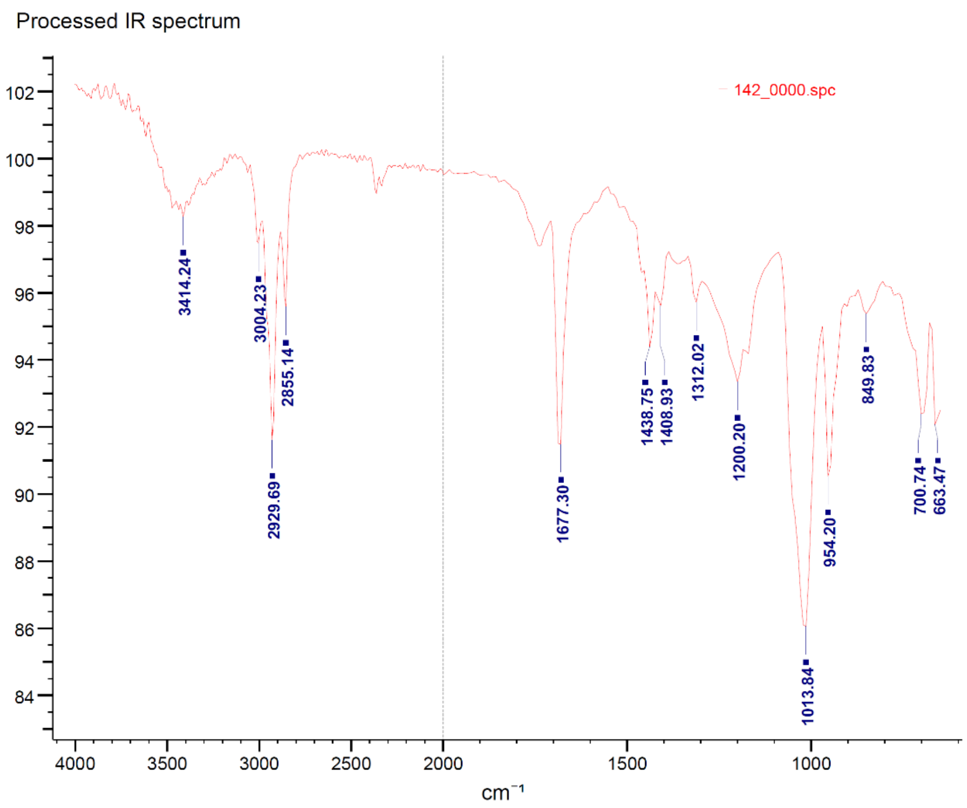

Figure 29. FT-IR spectrum of compound L2

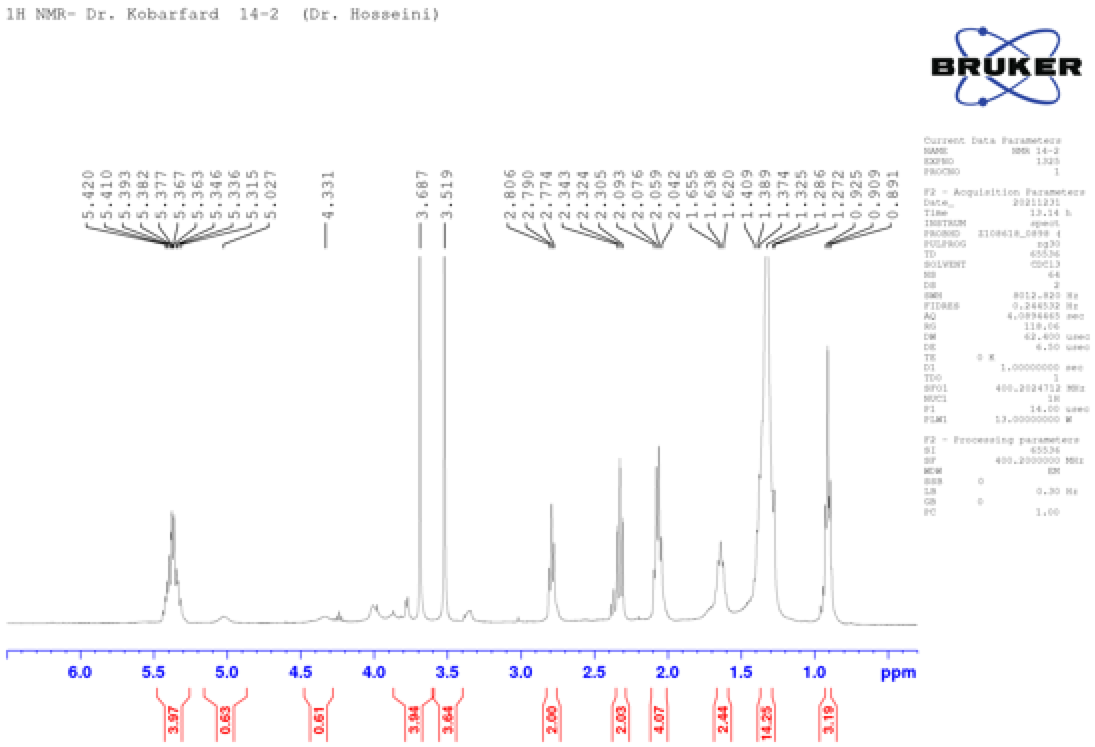

Figure 30. ^{1}H-NMR spectrum of compound L2

(9Z,12Z)-1-morpholinooctadeca-9,12-dien-1-one (L3)

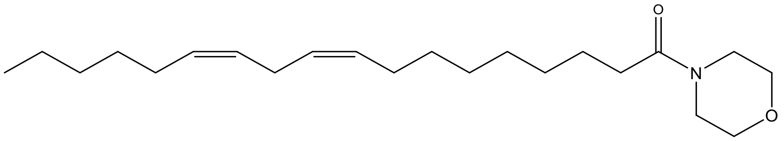

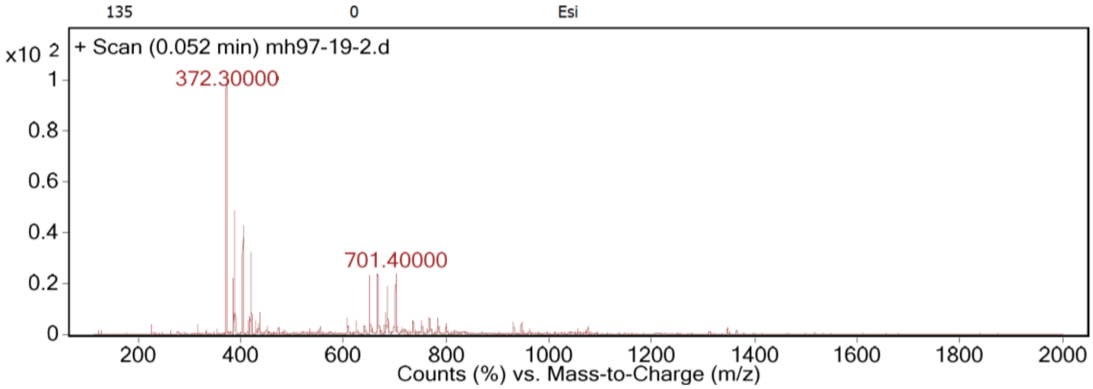

Figure 31. ESI-MS spectrum of compound L3

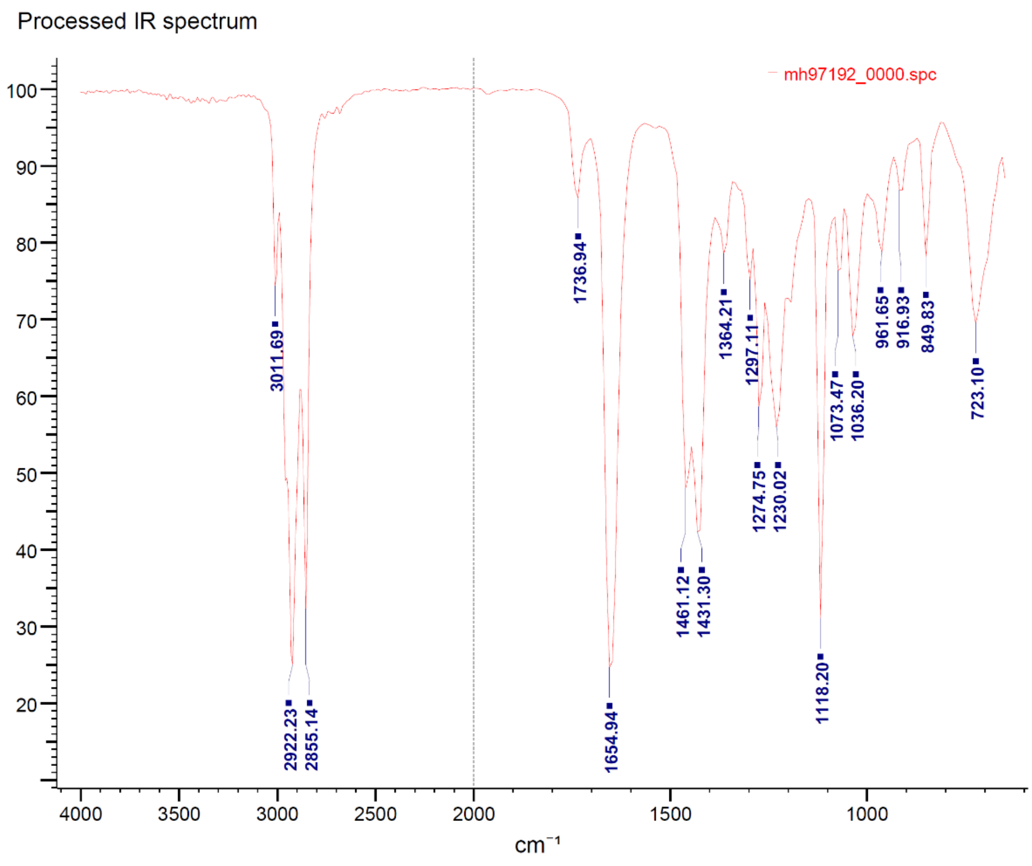

Figure 32. FT-IR spectrum of compound L3

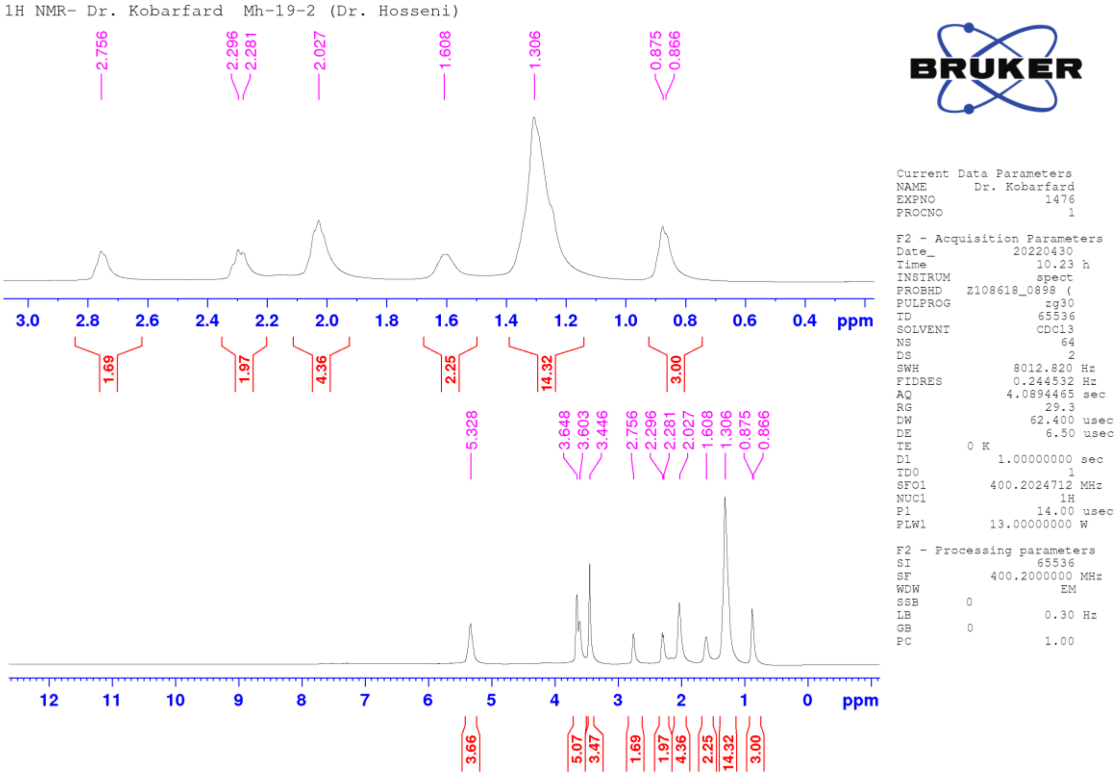

Figure 33. ^{1}H-NMR spectra of compound L3

(9Z, 12Z)-N-(4-hydroxyphenyl) octadeca-9,12-dienamide (L4)

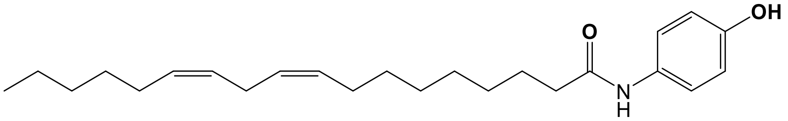

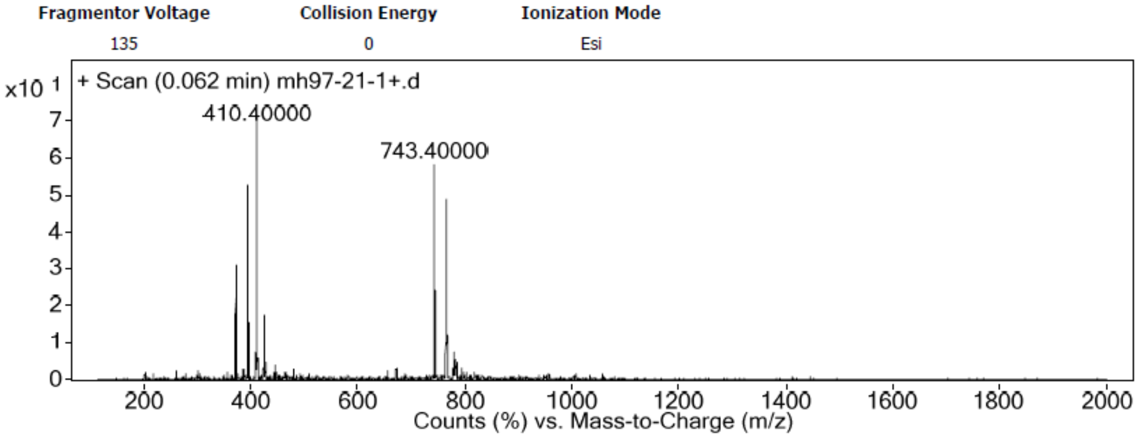

Figure 34. ESI-MS spectrum of compound L4

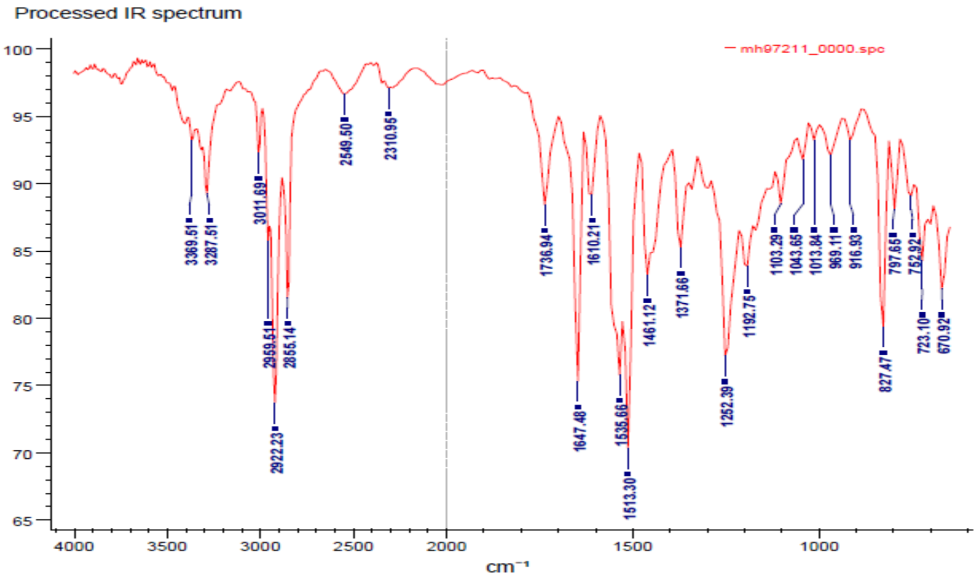

Figure 35. FT-IR spectrum of compound L4

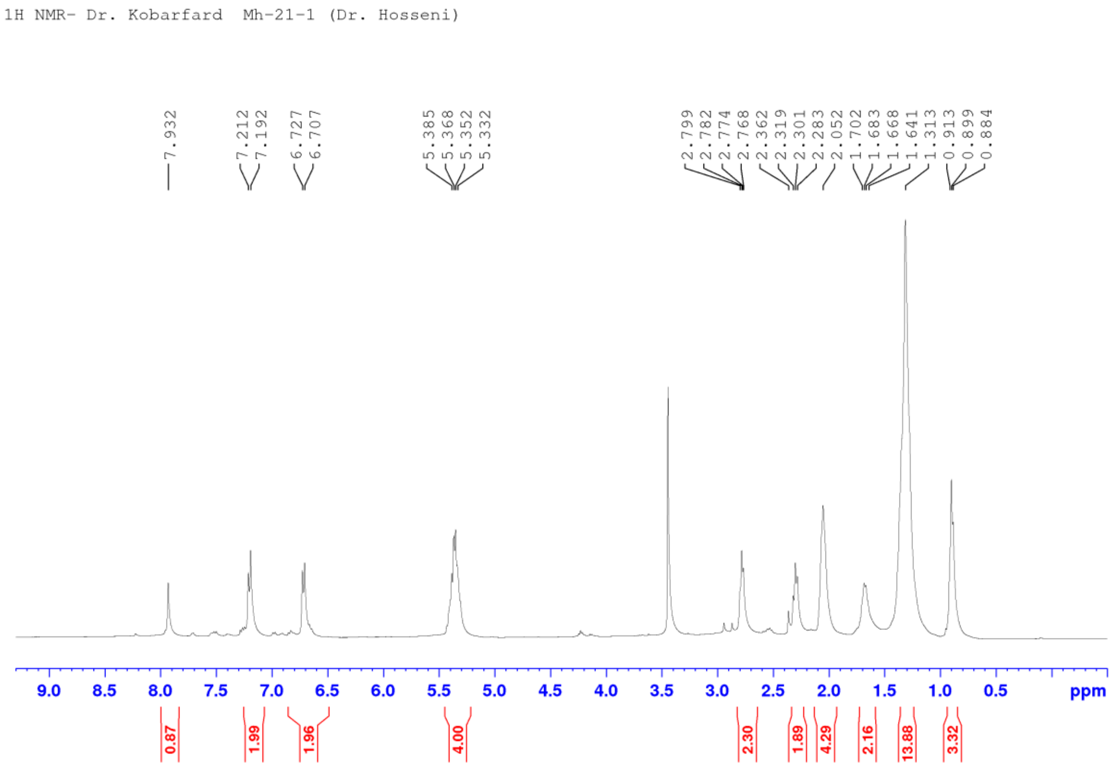

Figure 36. ^{1}H-NMR spectrum of compound L4

(9Z,12Z)-N-phenethyloctadeca-9,12-dienamide (L5)

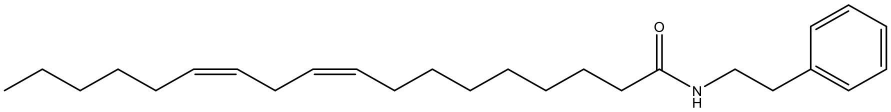

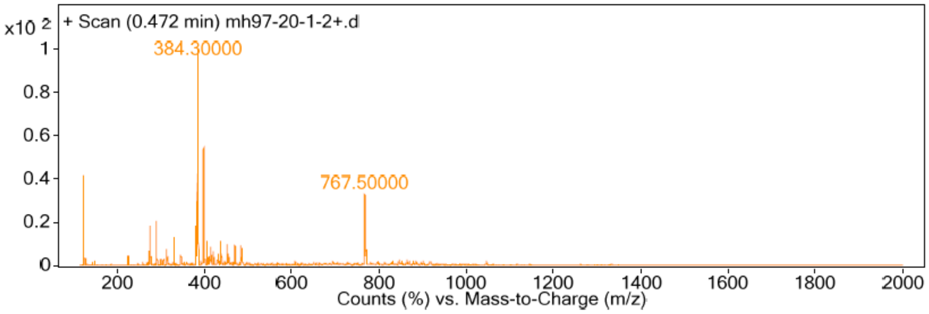

Figure 37. ESI-MS spectrum of compound L5

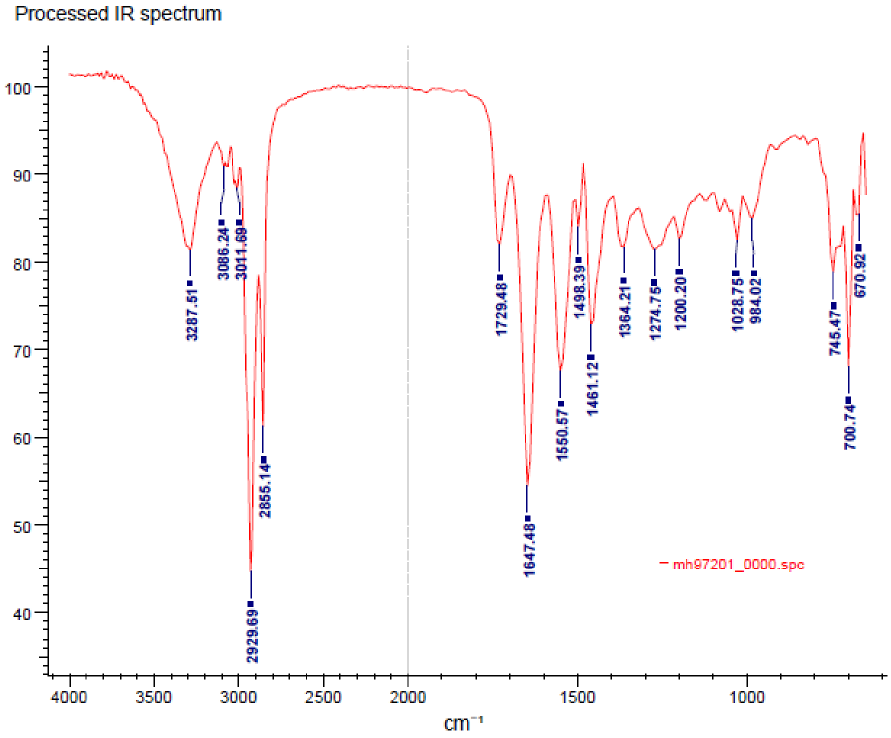

Figure 38. FT-IR spectrum of compound L5

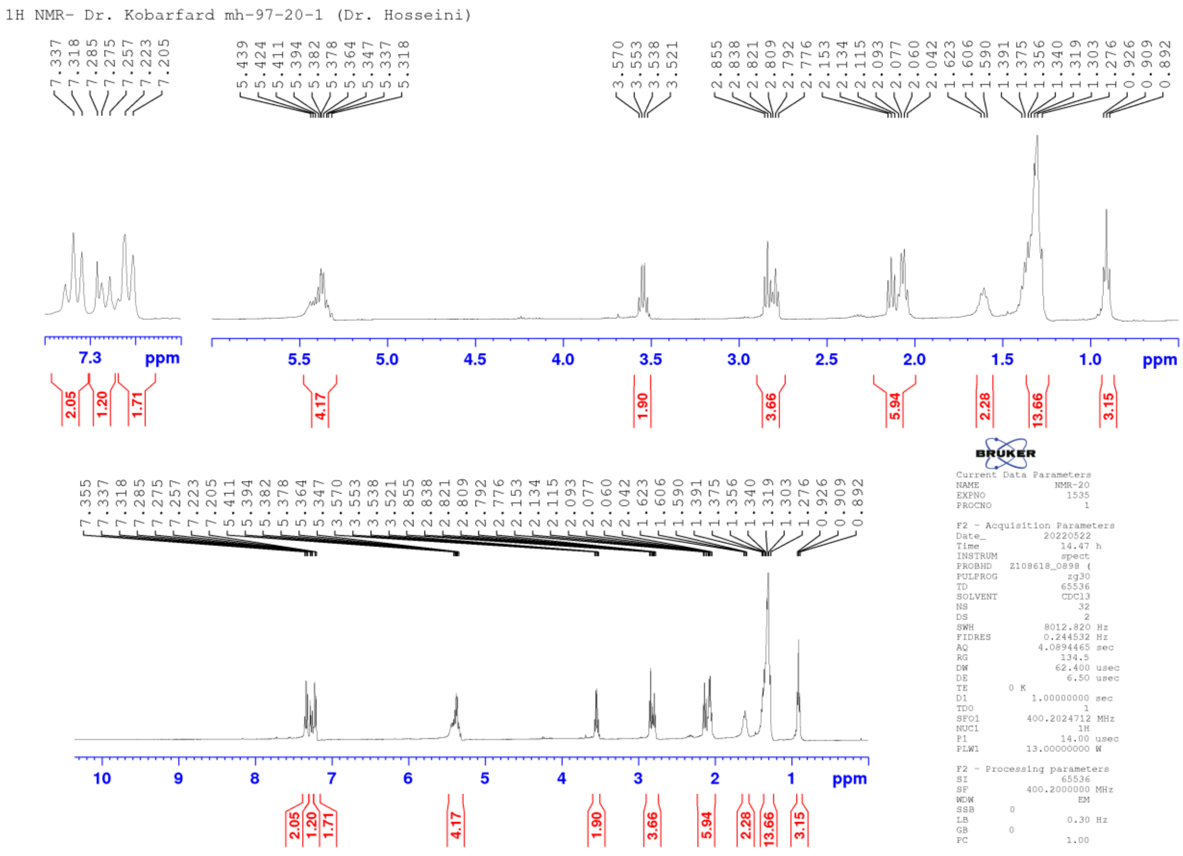

Figure 39. ^{1}H-NMR spectra of compound L5

(9Z,12Z)-1-(4-methylpiperazin-1-yl)octadeca-9,12-dien-1-one (L6)

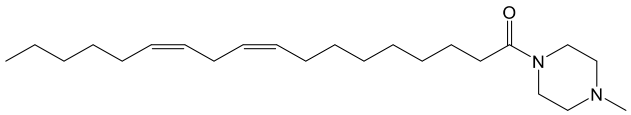

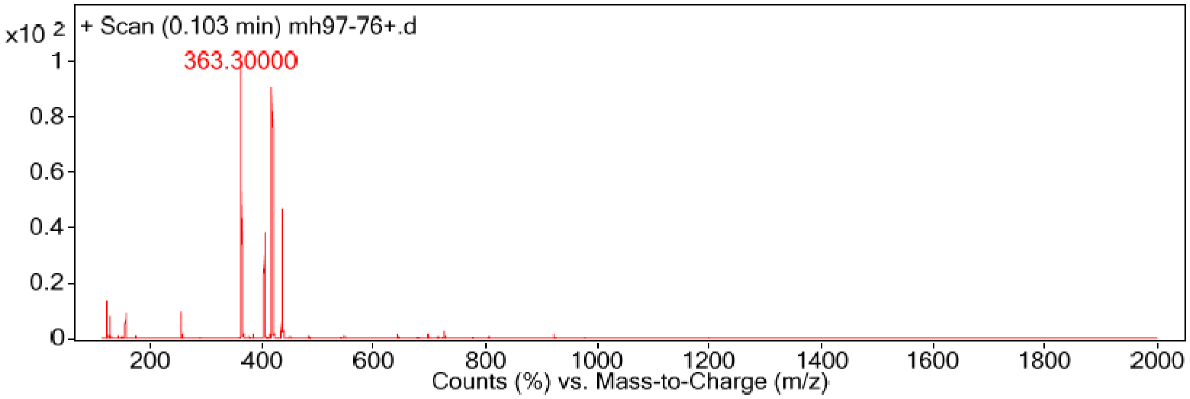

Figure 40. ESI-MS spectrum of compound L6

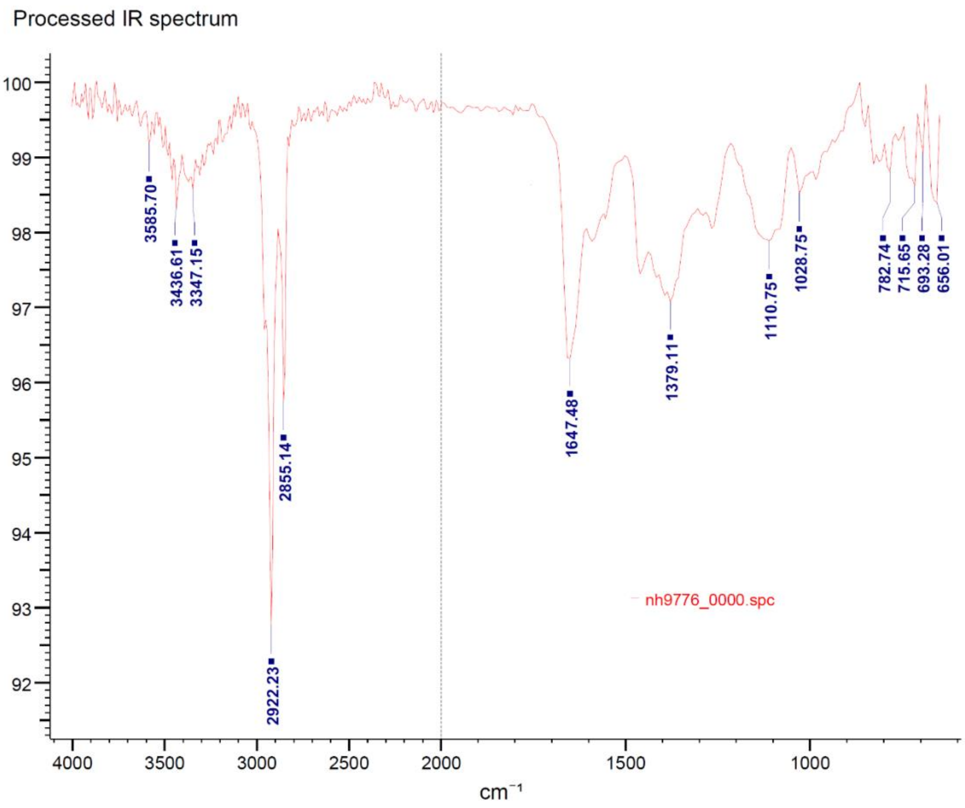

Figure 41. FT-IR spectrum of compound L6

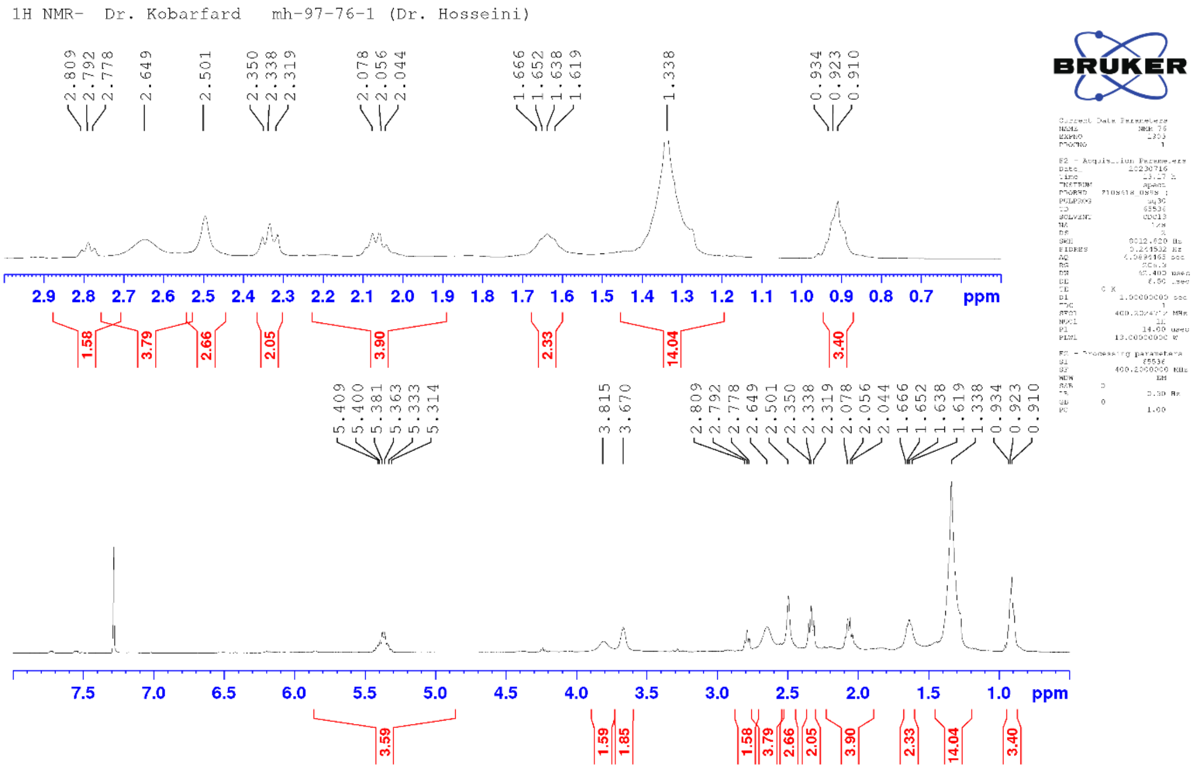

Figure 42. ^{1}H-NMR spectra of compound L6

(9Z,12Z)-N-(2,3-dihydroxypropyl)octadeca-9,12-dienamide (L7)

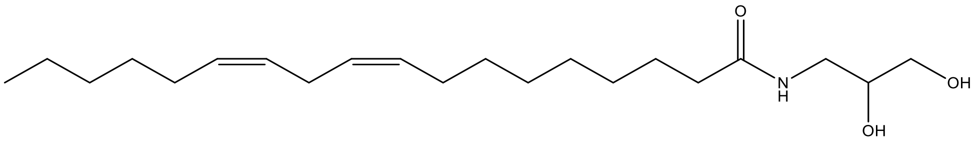

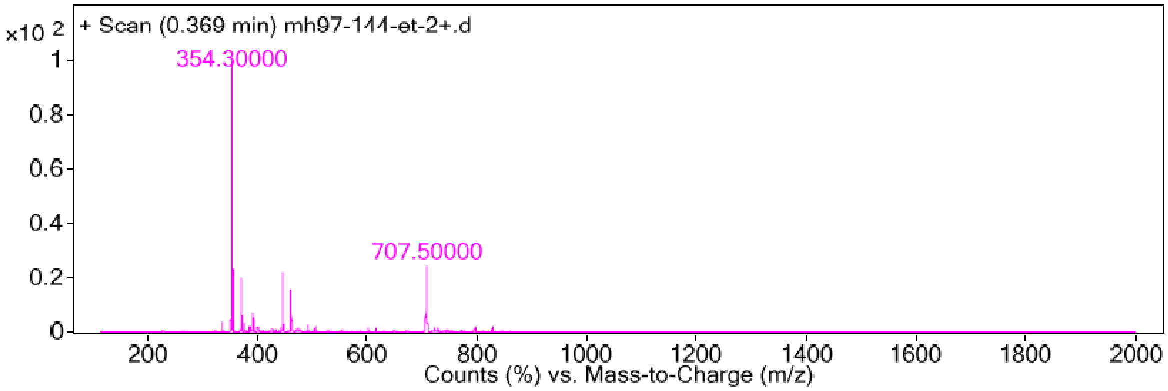

Figure 43. ESI-MS spectrum of compound L7 (m/z: 354 $(M+H)^{+}$)

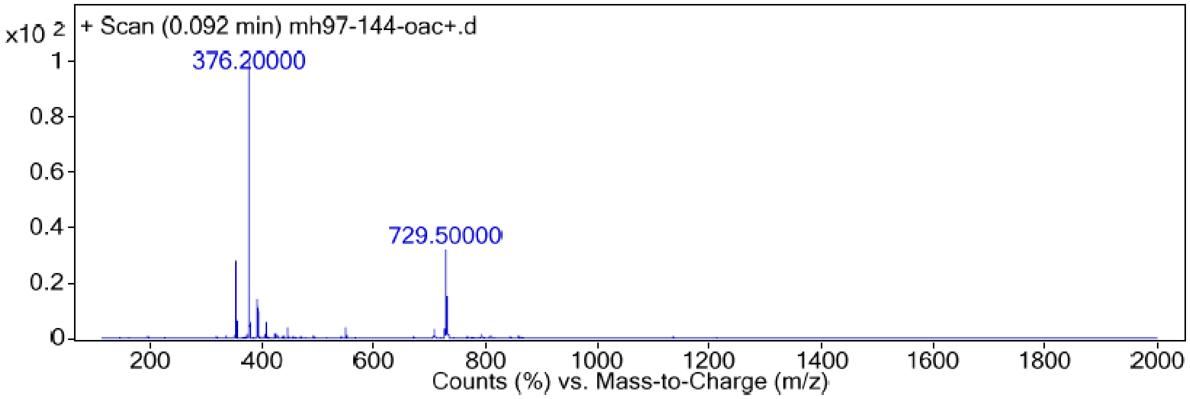

Figure 44. ESI-MS spectrum of compound L7 (m/z: 376 $(M+Na)^{+}$)

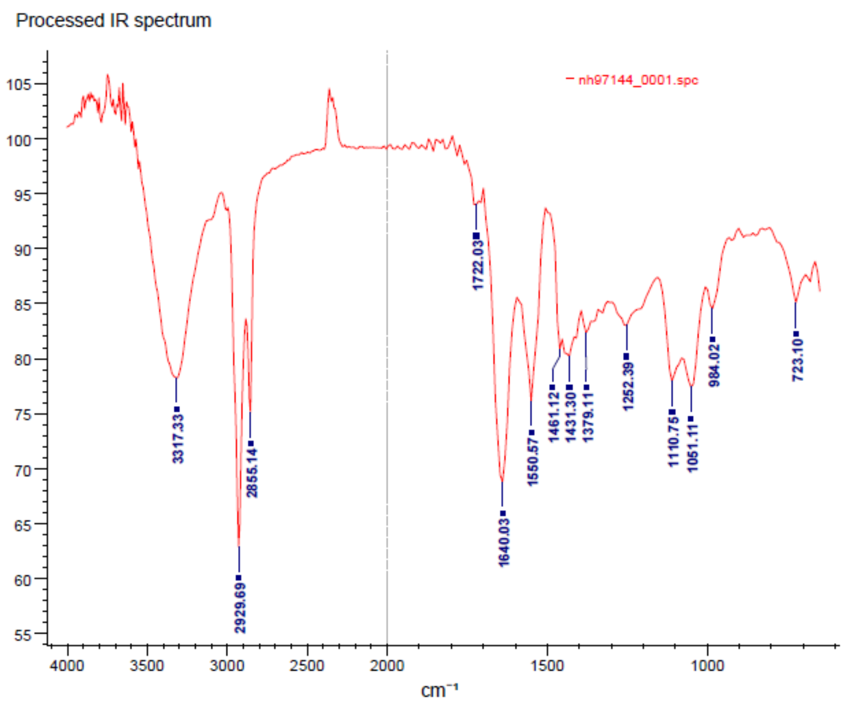

Figure 45. FT-IR spectrum of compound L7

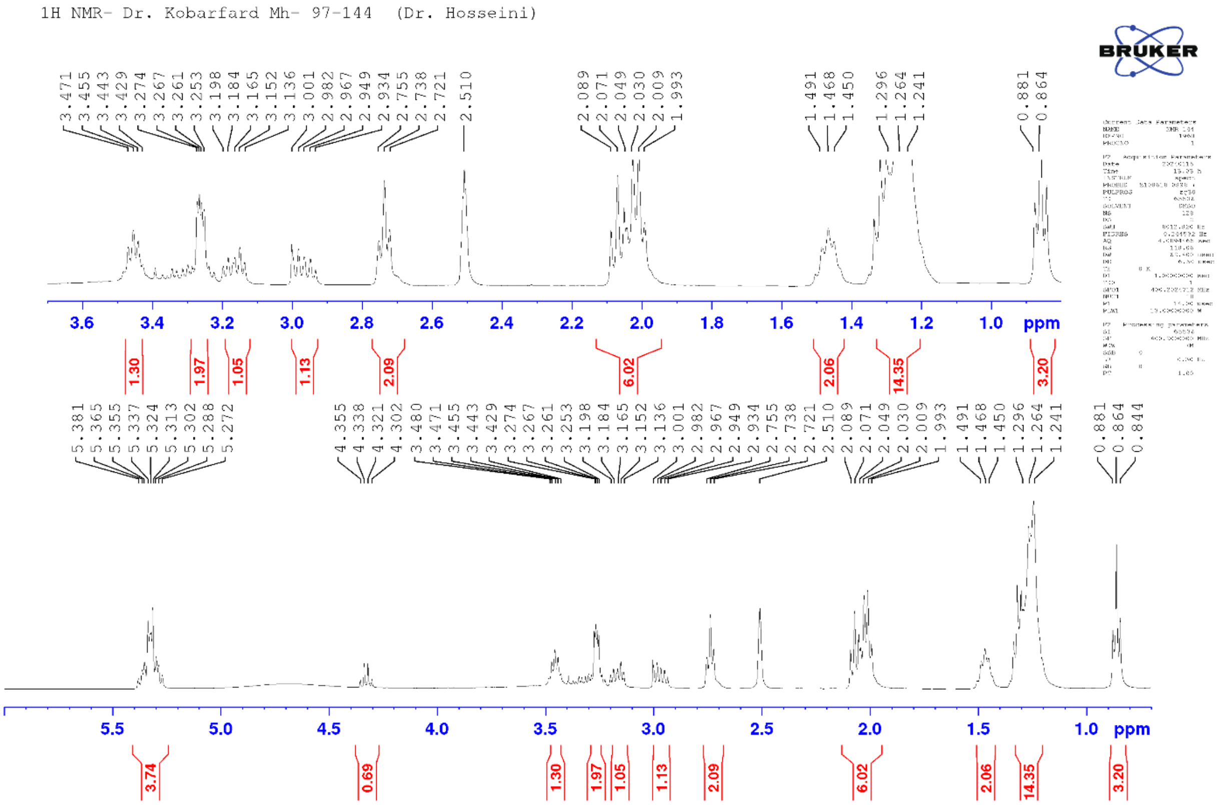

Figure 46. ^{1}H-NMR spectra of compound L7

(9Z,12Z)-N-(2-aminoethyl)octadeca-9,12-dienamide (L8)

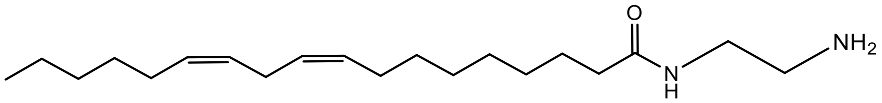

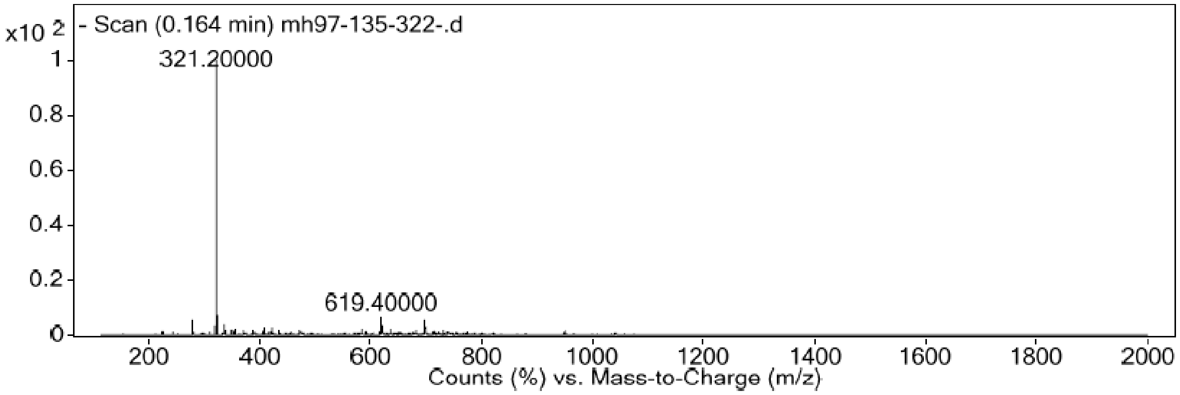

Figure 47. ESI-MS spectrum (negative mode) of compound L8

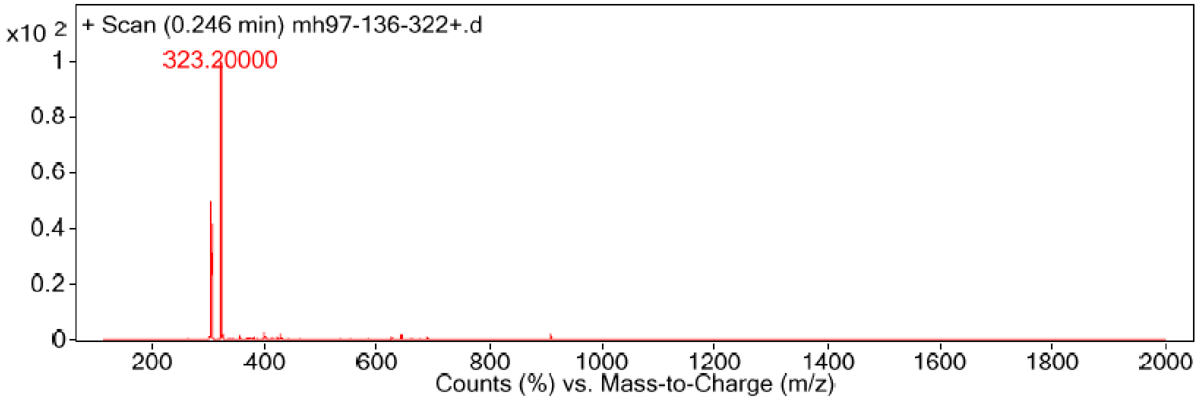

Figure 48. ESI-MS spectrum (positive mode) of compound L8

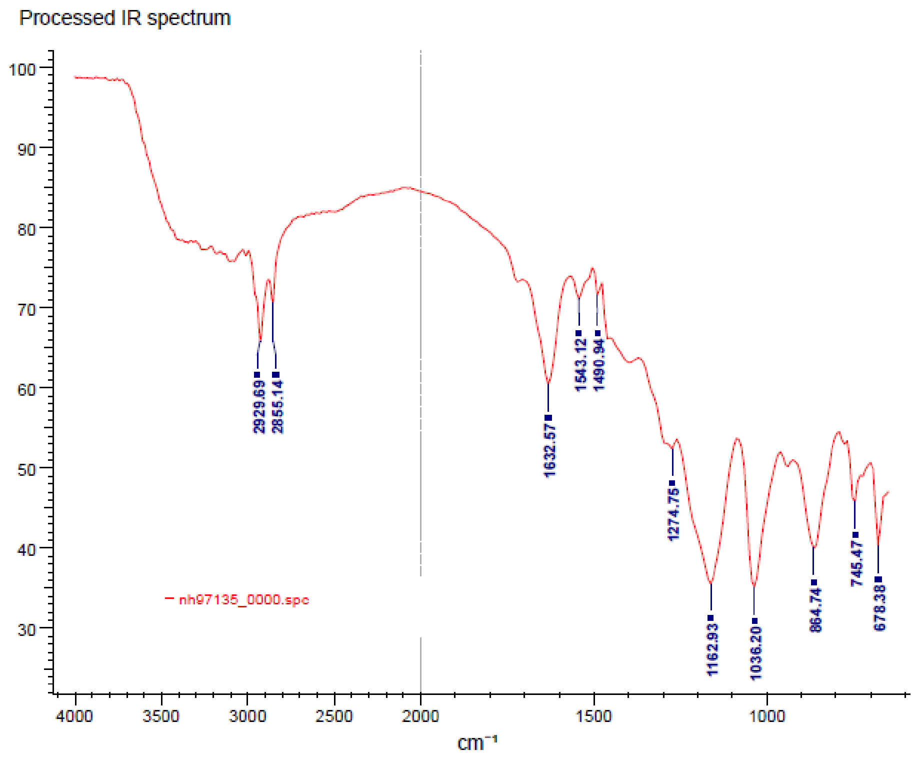

Figure 49. FT-IR spectrum of compound L8

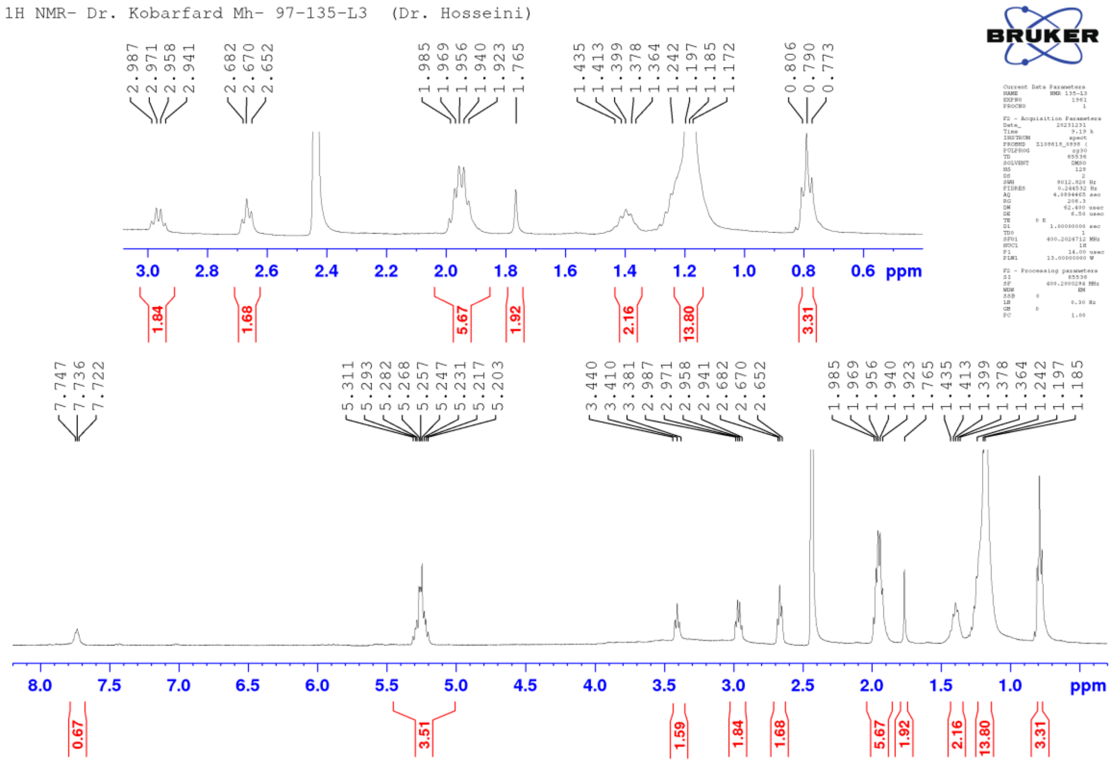

Figure 50. ^{1}H-NMR spectra of compound L8

Table 1: Binding affinity of the synthesized compounds.

Compound	Structure	Binding affinity (Kcal/mol)	
		1Q1M	1T4J
D1	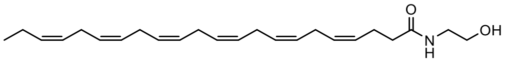	-6.3	-6.2
D2	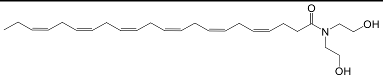	-6.2	-6.5
D3	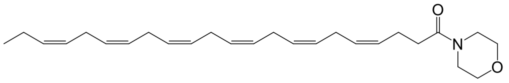	-6.0	-6.8
D4	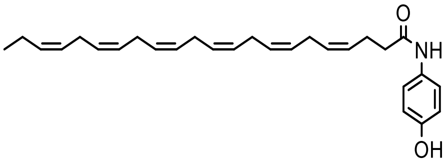	-6.5	-6.9
D5	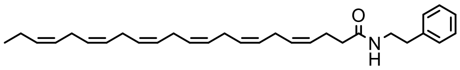	-6.1	-8.0
D6	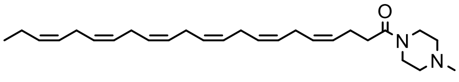	-5.7	-6.9
D7	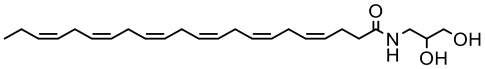	-5.7	-6.5
D8	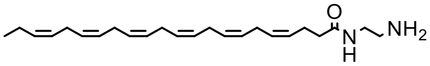	-5.5	-6.3
L1	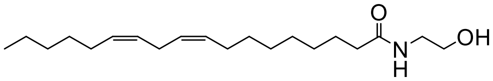	-5.4	-5.9
L2	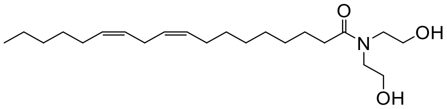	-5.8	-5.6
L3	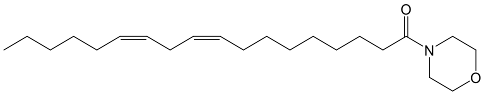	-5.3	-5.9
L4	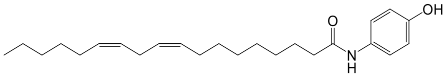	-6.0	-6.4
L5	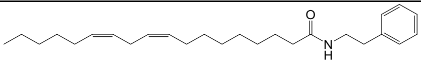	-6.0	-6.7

L6	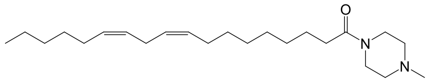	-6.3	-6.1
L7	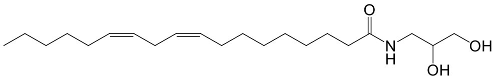	-5.7	-5.9
L8	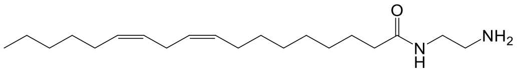	-5.1	-5.8
DHA	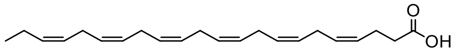	-6.3	-5.8
